# APOL1 risk alleles modulate T cell receptor signaling to promote allograft rejection

**DOI:** 10.1172/JCI193173

**Published:** 2026-06-09

**Authors:** John Pell, EM Tanvir, Zeguo Sun, Irene Chernova, Anand Reghuvaran, Soichiro Nagata, Mateus T. Guerra, John Choi, Soltan Al Chaar, Hiroki Mizuno, Ke Dong, Xin Tian, Reika Ishibe, Barbara Franchin, Paolo Cravedi, Ashwani Kumar, Gabriel Barsotti, Hongmei Shi, Bony De Kumar, Shinobu Smithson, Wenzhi Song, John Cijiang He, Anita S. Chong, Jordan S. Pober, Stefan Somlo, Ian W Gibson, Waldemar Popik, Zhongyang Zhang, Joseph Craft, Jamil Azzi, Naoka Murakami, Shuta Ishibe, Peter S Heeger, Madhav C. Menon

**Affiliations:** 1 Section of Nephrology, Yale University School of Medicine, New Haven, Connecticut, USA.; 2 Division of Nephrology, Department of Medicine, Icahn School of Medicine at Mount Sinai, New York, New York, USA.; 3 Section of Digestive Diseases, Department of Internal Medicine, Yale University School of Medicine, New Haven, Connecticut, USA.; 4 Transplant Research Center, Division of Renal Medicine, Brigham and Women’s Hospital, Harvard Medical School, Boston, Massachusetts, USA.; 5 Division of Nephrology, Washington University, St. Louis, Missouri, USA.; 6 Department of Surgery, The University of Chicago, Chicago, Illinois, USA.; 7 Department of Immunobiology, Yale University School of Medicine, New Haven, Connecticut, USA.; 8 Department of Pathology, University of Manitoba, Winnipeg, Manitoba, Canada.; 9 Center for AIDS Health Disparities Research, Department of Internal Medicine, Meharry Medical College, Nashville, Tennessee, USA.; 10 Cedars-Sinai Medical Center, Los Angeles, California, USA.

**Keywords:** Immunology, Nephrology, Transplantation

## Abstract

The exonic variants G1 and G2 in apolipoprotein-L1 (APOL1) are linked to an increased risk of kidney disease as well as kidney transplant rejection. Outside of the association of these prevalent variants with African ancestry, the underpinning causal mechanisms for rejection are unknown. We investigated T cell function using transgenic mice with physiologic expression of WT (G0), G1 APOL1 (G1), or G2 APOL1 (G2). Mice with the G1 or G2 variant showed greater CD8^+^ T cell activation with expansion of a central memory T cell (Tcm) subset. Stimulated G1 CD8^+^ T cells showed enhanced proliferation and cytokine production, which was reversed with APOL1 inhibition. In MHC-mismatched cardiac transplants, G1 mice demonstrated greater CD8^+^ T cell infiltration and worse survival. The bulk transcriptome of G1 CD8^+^ T cells and the single-cell transcriptome of graft-infiltrating Tcms showed enrichment of canonical T cell receptor (TCR) pathways including Ca^2+^ signaling. G1 CD8^+^ T cells demonstrated baseline ER Ca^2+^ depletion followed by sustained increases in cytosolic Ca^2+^ upon TCR stimulation. G1 CD8^+^ T cells were more sensitive to Ca^2+^ chelation, or store-operated Ca^2+^ entry inhibition, and were relatively resistant to calcineurin antagonism compared with G0 CD8^+^ T cells. Analogously, in a kidney transplant cohort, transplant recipients carrying an APOL1 risk variant (G1 or G2) who had elevated peripheral Tcms before transplantation developed rejection despite having significantly higher tacrolimus levels than recipients with the G0/G0 APOL1 genotype. In summary, we have unraveled an excitatory mechanism for APOL1 variants in T cells that causally links them to kidney rejection.

## Abstract 2

We show that APOL1 variants promote CD8+T-cell activation via Ca2+ signaling, causally explaining their link to kidney rejection.

## Introduction

African American (AA) individuals are at a disproportionate risk of focal segmental glomerulosclerosis (FSGS) because they carry the exonic variants G1 and G2 in the gene apolipoprotein L1 (*APOL1*) (1), which are seen exclusively in AA people or in those with admixed African ancestry (with the WT allele G0). APOL1 variants emerged as part of an “evolutionary arms race” against *Trypanosoma brucei* spp, which causes African trypanosomiasis (2). Following the discovery of FSGS-associated APOL1 variants (referred to hereafter as APOL1-RV for 1 risk variant or APOL1-RVs for 2 risk variants) (3), outstanding contributions have illuminated APOL1 FSGS. APOL1 is present only in some primates, limiting in vivo models (2). Elegant murine data showed that podocyte-specific overexpression of APOL1-RVs induces FSGS (4), and mechanistic clues regarding APOL1-induced cytotoxicity have been provided by non-mammalian (5) or rodent models (6, 7). APOL1 is a monovalent cation channel localized to organelle (5) and plasma (8) membranes, and increased channel activity in APOL1-RVs (vs. G0 APOL1) is probably a key mechanism for cytotoxicity in kidney epithelial cells (9), which are targeted by pharmacotherapeutics (10). Data from kidney transplantations demonstrate that APOL1-RVs in kidney donors increases the risk of death-censored allograft loss (DCAL) (11), which points to a kidney epithelial cell–centric role of APOL1-RVs in disease risk.

Provocative data from our laboratory suggested that the presence of APOL1-RVs in kidney transplant recipients is also associated with increased T cell–mediated rejection (TCMR) and DCAL (12), independent of the donor’s APOL1 genotype. Similar findings associating APOL1-RVs in kidney recipients with TCMR and DCAL have been reported in 2 independent studies (13, 14). Interestingly, recipient APOL1-RVs are associated with graft outcomes in an additive manner, i.e., every copy associated with increased risk (12, 14). Together, these data suggest a hitherto unexplored role for APOL1-RVs in T cells that are central to TCMR. However, even well-conducted epidemiologic studies cannot fully uncouple causation from association, especially since AA individuals (who carry APOL1-RVs) are also at higher risk of TCMR from multiple factors (14, 15). Therefore, defining the role of APOL1 variants in T cells using mechanistic models has significance for kidney transplants, given the high prevalence of this variant among AAs (~40% AAs have at least 1 copy of the risk variants).

To investigate the role and mechanism of APOL1-RVs in T cells, we generated bacterial artificial chromosome–transgenic (BAC-Tg) mice with the human *APOL1* promoter for G0, G1, and G2. BAC-Tg models result in physiologic levels of *APOL1*, as BAC inserts include *cis*-regulatory sequences to mirror endogenous transcription. Using this model, we report that APOL1-RV BAC-Tg mice show T cell activation and expansion of a central memory T cell (Tcm) subset. We then performed a series of mechanistic experiments incorporating interrelated translational human studies to identify a role and unravel the mechanism of APOL1-RVs in the context of T cell activation, allograft rejection, and graft survival.

## Results

### Generation and phenotyping of APOL1 BAC-Tg animals.

**Figure 1:**
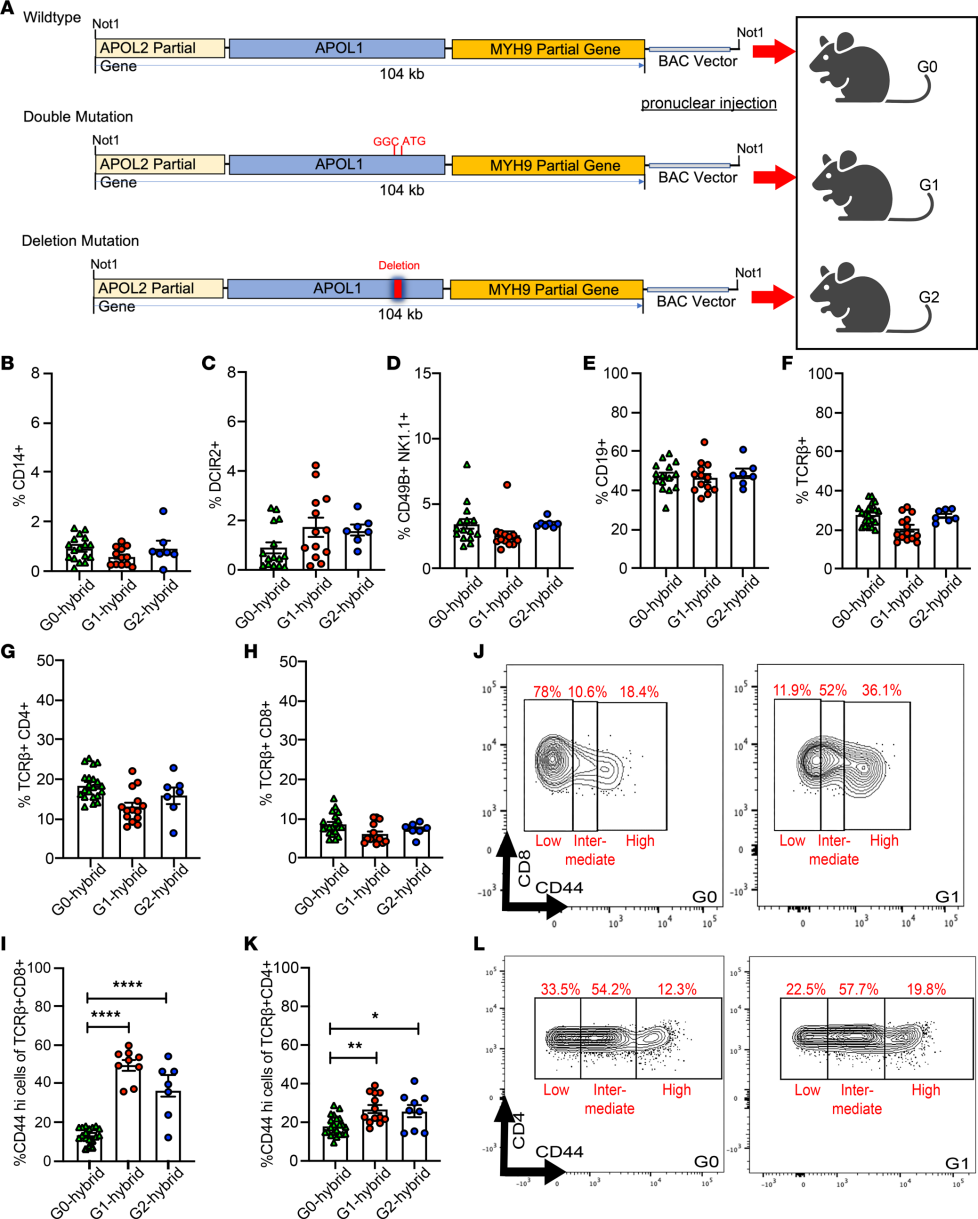
Development of APOL1 BAC-Tg mice and baseline T cell phenotypes. (**A**) APOL1 BAC-Tg mice were developed by pronuclear injection of a 104 kb BAC into oocytes to generate G0, G1, and G2 BAC-Tg mouse lines. (**B**–**H**) Baseline flow analysis of splenocytes comparing the percentages of innate immune cells (CD14^+^, DCIR^+^, CD49B^+^NK1.1^+^), as well as adaptive immune cells (B cells [CD19^+^], T cells [TCRβ^+^], CD4^+^, and CD8^+^ T cells) between the 3 lines. (**I**–**L**) Bar graphs of the percentages of (**I**) CD8^+^CD44^+^ and (**K**) CD4^+^CD44^+^ cells among T cells in spleens of BAC-Tg mice of the different lines at baseline. Plots in **J** and **L** are representative flow plots of data in **I** and **K**, respectively. Graphs show the mean ± SEM of 7 or more mice. *P < 0.05, **P < 0.01, and ****P < 0.0001, by Mann-Whitney *U* test. Individual mice are represented by circles (G1, G2) and triangles (G0).

APOL1-BAC-Tg mice (G0, G1, or G2 BAC-Tg) were generated as detailed in Figure 1A and Methods. Mice on a hybrid genetic background (G0, G1, and G2 hybrids) were obtained, and only mice with approximately 2 copy numbers of APOL1 BAC transgenes were used for the experiments (Supplemental Figure 1A; supplemental material available online with this article; https://doi.org/10.1172/JCI193173DS1). The expression of *APOL1* mRNA and APOL1 protein were confirmed in splenocyte lysates (Supplemental Figure 1, B and C).

We first studied splenocyte fractions by flow cytometry (flow) in G0, G1, and G2 hybrid mice (both sexes, 8–10 weeks old) to understand baseline immunologic phenotypes (*n* ≥7 mice each). The total splenocyte numbers were not significantly different between the BAC-Tg lines (Supplemental Figure 1D). The proportions of major innate immune cells (DCs [DCIR^+^], monocyte-macrophages [CD14^+^], NK cells [NK1.1^+^]) and adaptive immune cells (B cells [CD19^+^] and T cells [TCRβ^+^], CD4^+^ and CD8^+^ T cells) were similar across the genotypes (Figure 1, B–H, and Supplemental Figure 1, E and F for gating). On the basis of our prior human data showing T cell activation in individuals with APOL1 variants (12), we focused on T cell phenotype in BAC-Tg splenocytes using a characterized flow cytometric panel for T cell subsets (16). Interestingly, CD8^+^ and CD4^+^ T cells had a significantly increased proportion of the activation marker CD44 in G1 and G2 hybrid mice versus G0 hybrid mice at baseline (Figure 1, I–L, and Supplemental Figure 1G), with greater increases among CD8^+^CD44^+^ T cells versus G0 than among CD4^+^ T cells (Figure 1, I vs. K). CD8^+^CD69^+^ and CD4^+^CD69^+^ T cell proportions (early activation or tissue-resident marker) were not significantly different between G0, G1, or G2 hybrid mice at baseline (Supplemental Figure 1, H–K). In homeostasis at 12 weeks, APOL1-RV BAC-Tg mice showed no evidence of azotemia or albuminuria versus G0 BAC-Tg mice (Supplemental Figure 1, L and M). APOL1-RV BAC-Tg mice aged up to 1 year also showed no weight loss or alopecia (not shown).

### Variant APOL1 mouse splenocytes demonstrate increased CD8^+^ and CD4^+^ T cell activation at baseline in adult animals.

**Figure 2:**
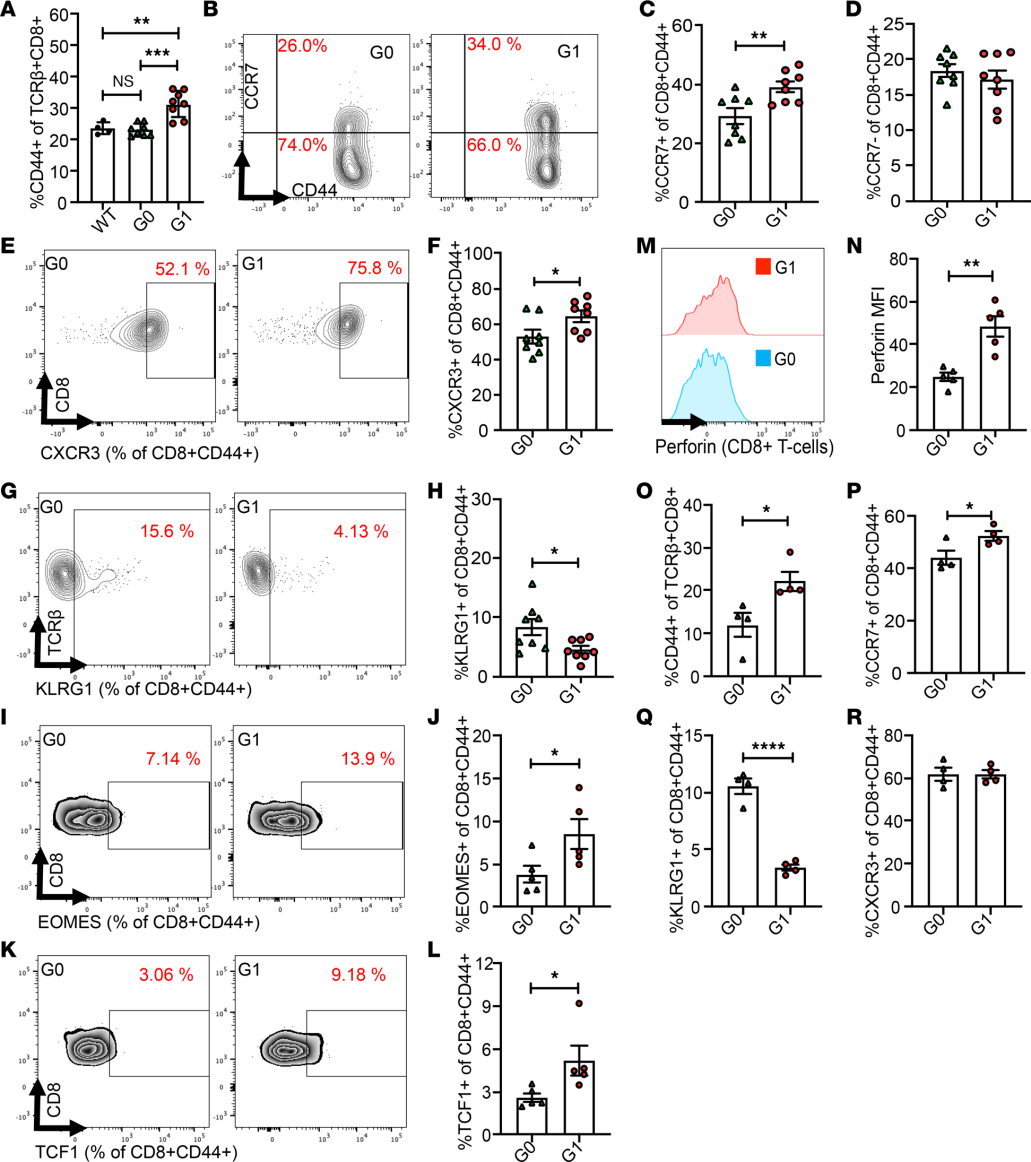
Baseline activation and polarization of CD8^+^ and CD4^+^ T cells in backcrossed variant APOL1 BAC-Tg mice. (**A**) Proportions of CD44^+^CD8^+^ T cells in G1, G0, and WT C57BL/6J mice. (**B**) Representative flow plots of CD8^+^CD44^+^ T cells gated for CCR7^+^ cells to identify Tcms. Proportions of (**C**) Tcms and (**D**) CD8^+^CD44^+^CCR7^–^ T cells from G1 versus G0 mice (*n* >5 mice each). (**E**–**H**) Representative flow plots and quantifications of the proportion of CD8^+^CD44^+^ T cells gated for (**E** and **F**) CXCR3^+^ T cells and (**G** and **H**) KLRG1^+^ T cells (*n* > 5 mice each). (**I**–**L**) Representative flow plots and quantification of cell proportions from intracellular staining of CD8^+^CD44^+^ T cells gated for the transcription factors (**I** and **J**) EOMES and (**K** and **L**) TCF1 (*n* = 5 mice each). (**M** and **N**) Representative flow plots and MFI of perforin in G0 and G1 mice. Mesenteric LN T cells were evaluated for proportions of (**O**) CD8^+^CD44^+^ T cells, (**P**) Tcms (CD8^+^CD44^+^CCR7^+^ T cells), (**Q**) CD8^+^CD44^+^KLRG1^+^ T cells, and (**R**) CD8^+^CD44^+^CXCR3^+^ T cells (*n* = 4 mice each). Bar graphs show the mean ± SEM. *P < 0.05, **P < 0.01, ***P < 0.001, and ****P < 0.0001, by Mann-Whitney *U* test. Circles and triangles represent individual mice.

Since the T cell phenotype is influenced by genetic background, we backcrossed our hybrid BAC-Tg lines with mice on a C57BL/6J background (B6) to generate G1 and G0 BAC-Tg B6 lines (hereafter referred to as G0 and G1) (Supplemental Figure 2A). APOL1 copy numbers were maintained through 6 backcrosses and showed no dilution of gene dose (Supplemental Figure 2B). We used G2 hybrid–Tg mice to validate selected findings. Among CD8^+^ T cells, CD44^+^ proportions remained higher in backcrossed G1 mice versus G0 or WT B6 mice, whereas G0 CD8^+^CD44^+^ T cell proportions did not differ from those in WT B6 mice (Figure 2A), suggesting gain of function with G1. Among CD8^+^CD44^+^ T cells, we observed significantly higher percentages of CD8^+^CD44^+^CCR7^+^ T cells (Tcms) (Figure 2, B and C), while CD8^+^CD44^+^CCR7^–^T cells were not increased (Figure 2D). Expansion of the Tcm subset in G1 BAC-Tg mice was not identifiable at weaning (4 weeks old) but was observed in all older mice (Supplemental Figure 2C) and in both sexes. We found that CD8^+^CD44^+^CXCR3^+^ T cells were increased in G1 BAC-Tg (Figure 2, E and F). Hence, G1 BAC-Tg mice had increased memory CD8^+^ T cells, specifically Tcms. Interestingly, CD8^+^CD44^+^KLRG1^+^ T cells were significantly reduced in G1 mice versus G0 mice (Figure 2, G and H). Intracellular transcription factor staining showed significant increases in the memory T cell marker EOMES (Figure 2, I and J) and the stemness marker TCF1 (Figure 2, K and L) in G1 CD8^+^CD44^+^ T cells. The proportions of BLIMP1^+^, TBET^+^, and RORγT^+^ cells were not different between G0 and G1 CD8^+^CD44^+^ T cells (Supplemental Figure 2, D–F). G1 CD8^+^ T cells also showed increased levels of the cytotoxicity marker perforin versus G0 T cells (Figure 2, M and N).

Among CD4^+^ T cells, we also observed significantly increased CD4^+^CD44^+^ T cells in G1 BAC-Tg mice (Supplemental Figure 2G); however, neither CXCR3^+^ nor CD4^+^CD44^+^CCR7^+^ T cell expansion was observed (Supplemental Figure 2, H and I). G1 CD4^+^CD44^+^ T cells also showed significantly higher TCF1 levels versus G0 T cells (Supplemental Figure 2J). EOMES^+^, BLIMP1^+^, TBET^+^, and RORγT^+^ cells were not different between G0 and G1 CD4^+^CD44^+^ T cells (Supplemental Figure 2, K–N). Th2 proportions (CD4^+^GATA3^+^) were also not different between G0 and G1 splenocytes (Supplemental Figure 2O). The proportions of Tregs (CD4^+^FOXP3^+^ T cells) were significantly reduced in pre-backcrossed G1mouose spleens (vs. G0) but were similar to those in G0 BAC-Tg mice after backcrossing (Supplemental Figure 2, P and Q).

Analysis of mesenteric lymph nodes (LNs) also confirmed increased CD8^+^CD44^+^ T cell proportions, Tcm expansion, and decreased CD8^+^CD44^+^KLRG1^+^ T cells but showed similar CD8^+^CD44^+^CXCR3^+^ T cell proportions in G1 versus G0 mice (Figure 2, O–R). No significant differences in activation or subset expansion were seen among mesenteric LN CD4^+^ T cells in G1 mice (Supplemental Figure 2, R–T). Fluorescence-minus-one plots used for gating key markers are shown in Supplemental Figure 2, U–X. Since activation and subset expansion were best demonstrated in CD8^+^ T cells, we focused our subsequent phenotyping experiments on CD8^+^ T cells.

Using PBMCs from patients with chronic kidney disease (CKD) (17), we evaluated the CD8^+^ T cell phenotype in individuals with or without APOL1-RVs (Supplemental Table 1 and Methods). The APOL1 genotype was confirmed from the electronic medical record or by Sanger sequencing by designing PCR probes to cover both the G1 and G2 alleles (SNP-IDs: rs73885319, rs60910145, rs71785313) (18). We identified significantly increased CD8^+^CXCR3^+^ and CD8^+^HLA-DR^+^ T cells in patients with APOL1-RVs versus those with G0/G0 alleles (Supplemental Figure 3, A–C). The proportion of CD8^+^CD127^+^CCR7^+^ Tcms tended to be higher but had wide variance (Supplemental Figure 3D). These human data support the CD8^+^ T cell activation phenotype seen in G1 BAC-Tg mice.

### Variant APOL1 CD8^+^ T cells show increased proliferation and central memory expansion following T cell receptor stimulation, which is reversed by an APOL1 inhibitor.

**Figure 3:**
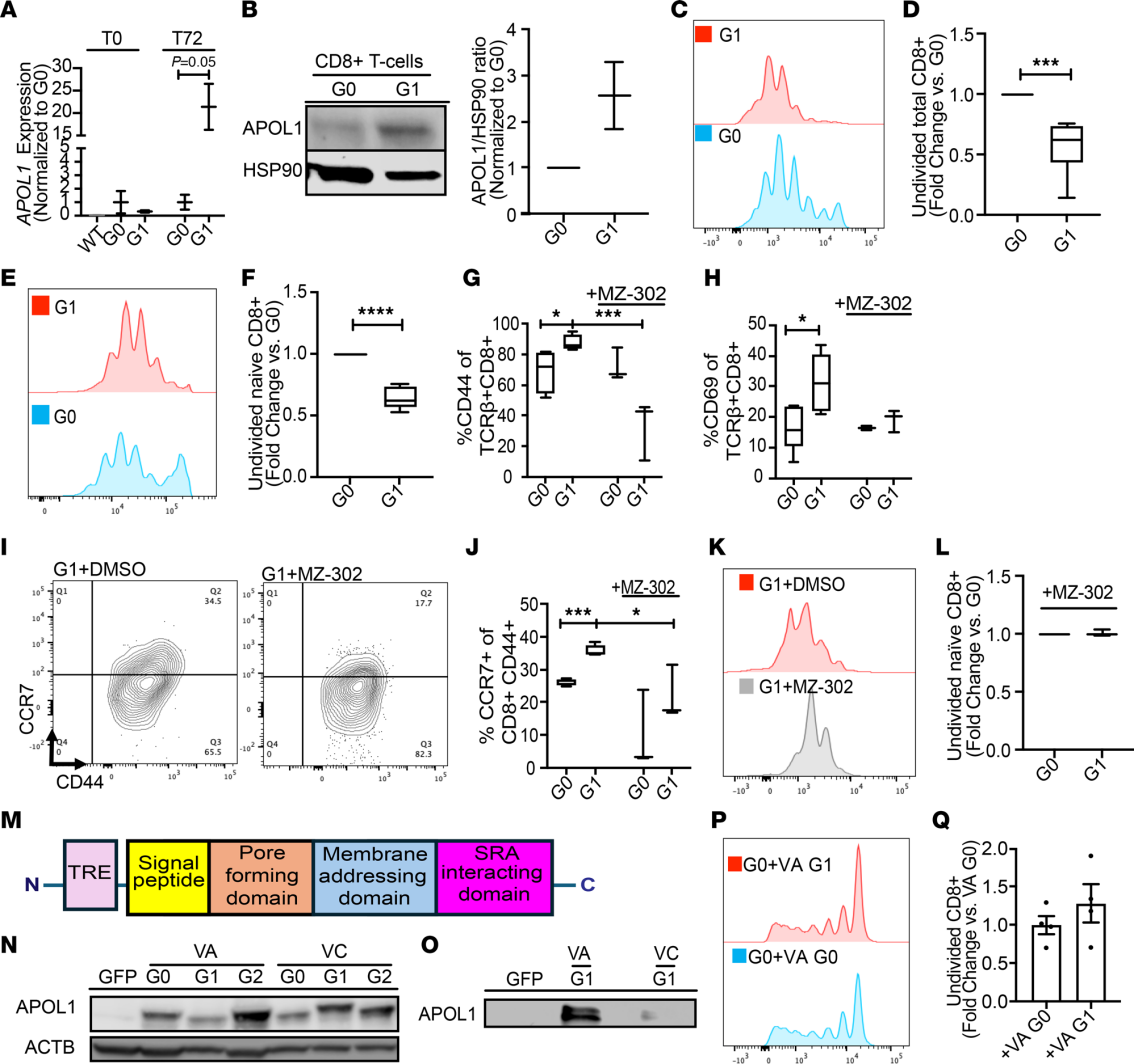
G1CD8^+^ T cells show increased proliferation with TCR stimulation that is reversed by APOL1 inhibition. (**A**) *APOL1* mRNA in naive G0, G1CD8^+^ T cells at baseline (time 0 hours [T0]) and after TCR stimulation (anti-CD3/anti-CD28, T72). (**B**) Representative APOL1 immunoblot and quantification from G0 and G1 CD8^+^ T cell lysates (*n* = 10 mice). (**C**) Proliferation plots and (**D**) fold changes of undivided total G0 and G1 CD8^+^ T cells after TCR stimulation. (**E** and **F**) Identical data from naive G0 and G1 CD8^+^ T cells. (**G**–**J**) G0 or G1 CD8^+^ T cells were TCR stimulated with or without MZ-302. (**G**) Proportions of CD8^+^CD44^+^, (**H**) CD8^+^CD69^+^, and (**J**) CD8^+^CD44^+^CCR7^+^ T cells and (**I**) representative flow plots. (**K**) Proliferation plots and (**L**) quantification of undivided naive G0 and G1 CD8^+^ T cells after MZ-302 (APOL1 inhibitor) treatment. Box-and-whisker plots show the median and range of triplicates pooled from more than 5 mice each. (**M**) APOL1 protein/functional domains. Immunoblots from (**N**) cell lysates and (**O**) supernatants of HEK293 T cells overexpressing APOL1 protein (VA-G0, -G1, -G2) or deletion constructs (VC-G0, -G1, -G2) probed for APOL1/ACTB. (**P**) Proliferation plots of G0 CD8^+^ T cells treated with VA-G1 or VA-G0 supernatant. (**Q**) Bar graphs show fold changes of undivided CD8^+^ T cells (*n* = 4 each). *P < 0.05, ***P < 0.001, and ****P < 0.0001, by paired, 2-tailed *t* test for fold change and Mann-Whitney *U* test for percentages.

PBMCs from healthy individuals had shown increased *APOL1* expression after T cell receptor (TCR) stimulation of naive CD8^+^ T cells (12). To study T cell function with APOL1-RVs, we isolated CD8^+^ T cells from G0 and G1 mice (see Methods) and stimulated them with anti-CD3/anti-CD28 antibodies (Supplemental Figure 4A). We found that *APOL1* expression was similar in naive G0 and G1 CD8^+^ T cells, but proliferated G1 T cells had significantly higher *APOL1* expression levels (Figure 3A). Consistent with this observation, total CD8^+^ T cells from G1 mice (with a greater proportion of CD8^+^CD44^+^ T cells) showed increased APOL1 versus G0 CD8^+^ T cells (Figure 3B). Ex vivo–stimulated G1 CD8^+^ T cells showed enhanced proliferation compared with G0 CD8^+^ T cells (Figure 3, C and D). Naive T cells from G1 and G0 spleens were isolated and stimulated ex vivo (Supplemental Figure 4, A and B). Ex vivo–stimulated naive G1 or G2 CD8^+^ T cells also showed significantly increased proliferation (Figure 3, E and F, and Supplemental Figure 4, C and D) versus G0 CD8^+^ T cells. TCR-stimulated naive G1 CD8^+^ T cells showed increased CD69^+^ T cells (recently activated) and increased CD44^+^ T cell proportions with expansion of Tcms (Figure 3, G–J). This was accompanied by reduced viability and increased annexin V positivity in G1 CD8^+^ T cells (Supplemental Figure 4, E–H). Altered proliferation, activation, expansion, viability, and apoptosis in G1 CD8^+^ T cells was mitigated by MZ-302, an APOL1 inhibitor (Figure 3, G–L, and Supplemental Figure 4, E–H) (19). MZ-302 had no effect on the activation or proliferation of WT-B6 CD8^+^ T cells without *APOL1* (Supplemental Figure 4, I and J).

APOL1 is the only secreted member among APOL proteins, and we evaluated the role of secreted APOL1 in T cell activation. We generated lentiviral, doxycycline-inducible (DOX-inducible) APOL1-overexpressing constructs with either complete sequences (VA-G0, VA-G1) or with a deletion of the 21 amino acid signal peptide (VC-G0, VC-G1) (Figure 3M). In HEK293T cells, inducible overexpression of these constructs was confirmed in immunoblots of cell lysates after 24 hours of overexpression (Figure 3N). Supernatant immunoblotting confirmed that only VA construct–expressing, but not VC construct–expressing, cells had secreted APOL1 protein (Figure 3O). We next induced proliferation of G0 T cells using anti-CD3/anti-CD28 antibodies in the presence of supernatant from HEK293 T cells overexpressing either VA-G1 or VA-G0 APOL1. The proliferation and viability of G0 T cells were unchanged by the addition of secreted G1 or G0 APOL1 (Figure 3, P and Q, and Supplemental Figure 5, A–C). Furthermore, induction of *APOL1* (with DOX) showed reduced viability in VA-G1 and VC-G1 cell lines compared with the corresponding G0 lines (Supplemental Figure 5D). Together, these data demonstrate an excitatory effect of APOL1-RVs in T cells upon TCR stimulation and that the secreted APOL1 variant plays a minimal role in the T cell proliferation phenotype.

### Variant BAC-Tg T cells produce excess proinflammatory cytokines and phenocopy patients with APOL1-RVs following immune or viral stimulation.

**Figure 4:**
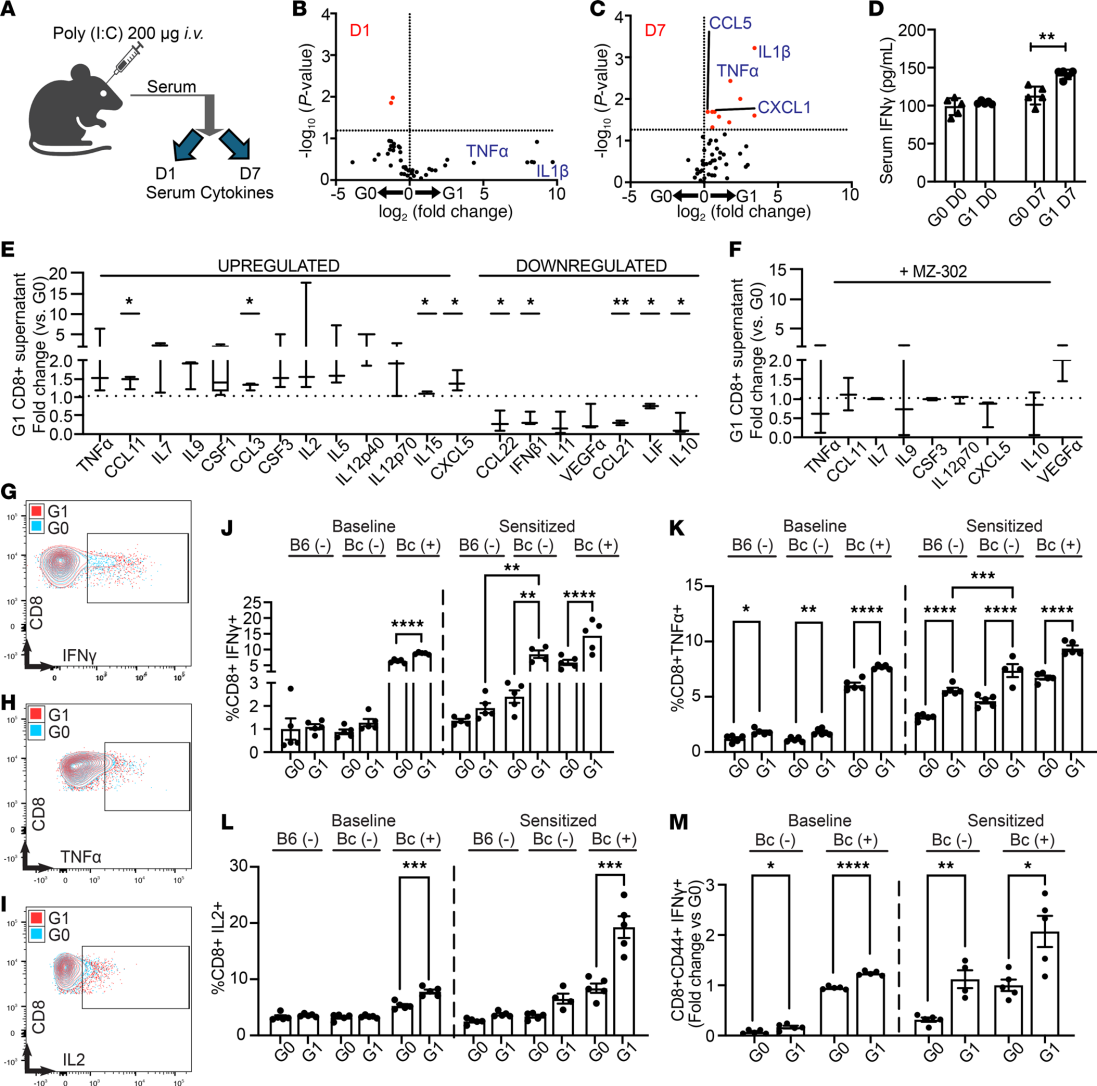
CD8^+^ T cells and sera from variant BAC-Tg mice demonstrate cytokine excess following immune or viral stimulation. (**A**) Schema of the Poly(I:C) study. (**B**) Volcano plots of multiplex serum cytokine profiles at day 1 (D1) and (**C**) day 7 (D7) following Poly(I:C). The horizontal dotted line corresponds to *P* = 0.05 (unpaired, 2-tailed *t* test). (**D**) Serum IFN-γ levels by ELISA in G1 and G0 mice at day 0 and day 7 after Poly(I:C). (**E** and **F**) Box-and-whisker plots showing (**E**) G1 CD8^+^ T cell supernatant cytokine level fold changes (normalized to G0) and (**F**) with MZ-302 inhibitor treatment. (**G**–**I**) Representative flow plots of (**G**) IFN-γ, (**H**) TNF-α, and (**I**) IL-2 production by BAC-Tg CD8^+^ T cells from G1 versus G0 mice after 10 days of sensitization with BALB/c splenocytes (Bc). (+) indicates treatment also with PMA and ionomycin. (**J**–**L**) Bar graphs quantify the respective cytokine production levels both before and after sensitization in BAC-Tg CD8^+^ T cells from G1 versus G0 mice (*n* = 5 mice each), whereas (**M**) quantifies IFN-γ production levels within CD44^+^CD8^+^ T cells in these same animals. B6 splenocyte stimulation with or without PMA and ionomycin [(+) or (–)] is shown as the control. Graphs show the mean ± SEM. *P < 0.05, **P < 0.01, ***P < 0.001, and ****P < 0.0001, by Mann-Whitney *U* test. Circles and triangles represent individual mice.

**Figure 5:**
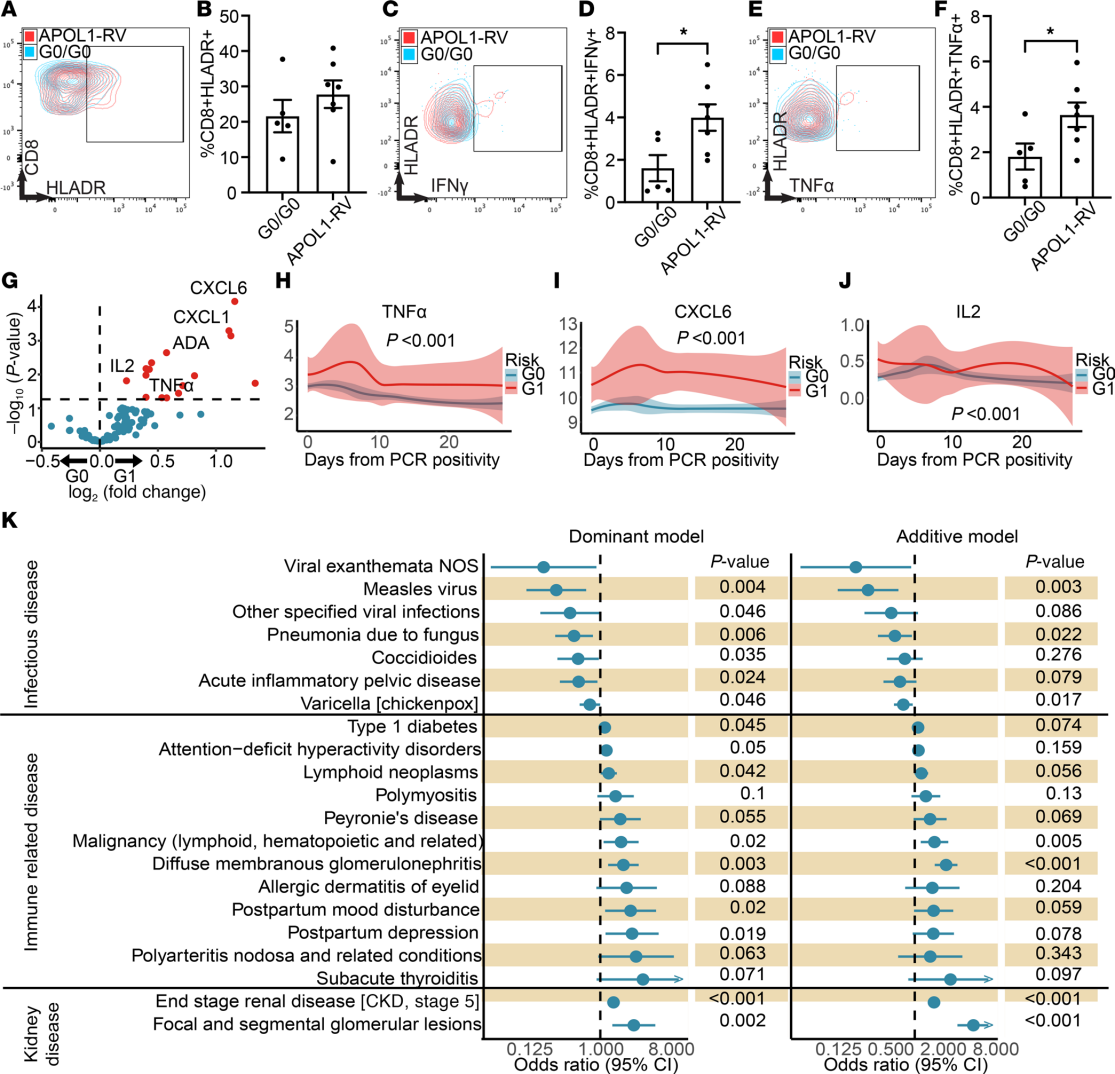
Human CD8^+^ T cells and sera from patients with APOL1-RVs phenocopy the cytokine excess observed in BAC-Tg mice following immune or viral stimulation. (**A**–**F**) Representative flow plots of (**A**) HLA-DR, (**C**) IFN-γ, and (**E**) TNF-α expression. (**B**, **D**, and **F**) Bar graphs show the corresponding quantifications from human APOL1-RV or G0/G0 T cells in CKD cohorts (*n* = 7 vs. 5; see Supplemental Table 1 and Methods). (**G**–**J**) Volcano plot (**G**) of cytokine expression changes during COVID-19 in G1 versus G0 participants from the CHARM study. Corresponding line graphs of serum (**H**) TNF-α, (**I**) CXCL6, and (**J**) IL-2 levels (*x* axis shows time: days 0–28 from PCR positivity; *y* axis shows normalized protein expression by Olink [mean ± 95% CI = line/shaded area]). (**K**) Epidemiologic data from the All-of-Us cohort using PheWAS after adjustment for sex and genetic ancestry. Forest plot shows selected phenotype associations with the APOL1-RV genotype as dominant or additive models (shown as OR with 95% CI). Graphs show the mean ± SEM. *P < 0.05, by Mann-Whitney *U* test. Dots represent individual patients.

To study cytokine production in response to viral infections in G1 BAC-Tg mice, we injected the viral nucleic acid mimic Poly(I:C) intravenously into G0 and G1 BAC-Tg mice then profiled serum cytokines (on days 1 and 7; Figure 4A). Multiplex cytokine profiling of sera from G1 mice showed upregulation of proinflammatory cytokines starting from day 1, which significantly increased by day 7 (11 of 45 significantly increased in G1 serum vs. 0 of 45 significantly increased in G0 serum; Figure 4, B and C). IFN-γ was increased in G1 serum by ELISA at day 7 (Figure 4D). Since Poly (I:C) stimulates TLR3 (20), and both TLR and TCR stimulation co-occur during viral infections, we tested cytokine excess after TCR stimulation. In this study, we first performed cytokine profiling of supernatants from ex vivo–anti-CD3/anti-CD28–stimulated G1 or G0 CD8^+^ T cells. G1 T cell supernatant showed upregulation of 13 of 44 cytokines, whereas 7 of 44 cytokines were downregulated in 3 independent replicate experiments (*n* >5 mice/experiment; Supplemental Table 2). TNF-α and IL-2 proinflammatory cytokines with TCR activation were upregulated, whereas immunoregulatory cytokines, including IL-10 and VEGF, were downregulated (Figure 4E). Treatment with MZ-302 (10 μM) reversed both the up- and downregulation of cytokines in G1 versus G0 T cell supernatants (Figure 4F). For allostimulation, we used irradiated BALB/c splenocytes to stimulate BAC-Tg CD8^+^ T cells from mice with or without prior BALB/c splenocyte sensitization (day 10). G1 BAC-Tg CD8^+^ T cells showed significantly increased IFN-γ, TNF-α, and IL-2 levels after sensitization and increased IFN-γ levels even in unsensitized mice (Figure 4, G–M). Hence, G1 BAC-Tg CD8^+^ T cells expressing proinflammatory cytokines demonstrated enhanced responses to allosensitization or viral stimuli. We also stimulated human PBMCs from the Yale CKD cohort (Supplemental Table 1) with anti-CD3/anti-CD28 (along with Golgi block). At 24 hours, activated CD8^+^ T cells from individuals with APOL1-RVs (HLA-DR^+^CD8^+^) produced more IFN-γ and TNF-α than did CD8^+^ T cells from individuals with the G0/G0 genotype (Figure 5, A–F).

In humans, COVID-19 is a viral infection associated with systemic cytokine excess and APOL1-induced disease (21, 22). To understand whether cytokine excess is also identifiable in APOL1-RV patients with COVID-19, we utilized the COVID-19 Health Action Response for Marines (CHARM) study in which previously healthy members of the United States Marine Corps underwent serial sera collection before, during, and after SARS-CoV-2 infection (Table 1). These study participants were minimally symptomatic. We confirmed the APOL1 genotype using RNA-seq data (12). Serum cytokine trends were analyzed (96-plex Olink) using linear mixed models to incorporate multiple time points for each CHARM study participant (see Methods). Individuals with the G1 variant showed significantly higher levels of 35 of the 96 cytokines (*P* < 0.05) (any G1 vs. G0/G0, *n* = 6 vs. 50) (Figure 5G and Supplemental Table 3). Similarly, 20 of 96 cytokines were significantly elevated in the US Marine Corps participants with any variant (i.e., any G1 or G2 [*n* = 12] vs. G0/G0 [*n* = 50]; Supplemental Figure 6A and Supplemental Table 4). APOL1-RV sera also showed persistence of elevated cytokine levels (Figure 5, H–J, and Supplemental Figure 6, B–D), altogether demonstrating relevance in humans of increased cytokine responses with APOL1-RVs during viral infection.

We also asked whether APOL1-RV status has epidemiologic associations with viral infections or immunologically mediated diseases by probing the All-of-Us cohort for genotype-phenotype associations (i.e., phenome-wide association study [PheWAS]; see Methods). In this cohort, aside from the clear association with renal diseases with APOL1-RVs, we observed significant associations of a reduced risk of viral/fungal infectious diseases, but an increased risk of immunopathogenetic conditions with APOL1-RV status in both dominant and additive models (Figure 5K), aligning with a potential immunologic and antimicrobial role for APOL1 variants outside of transplantation.

### Variant APOL1 BAC-Tg mice demonstrate accelerated allograft rejection in heart transplant models.

**Figure 6:**
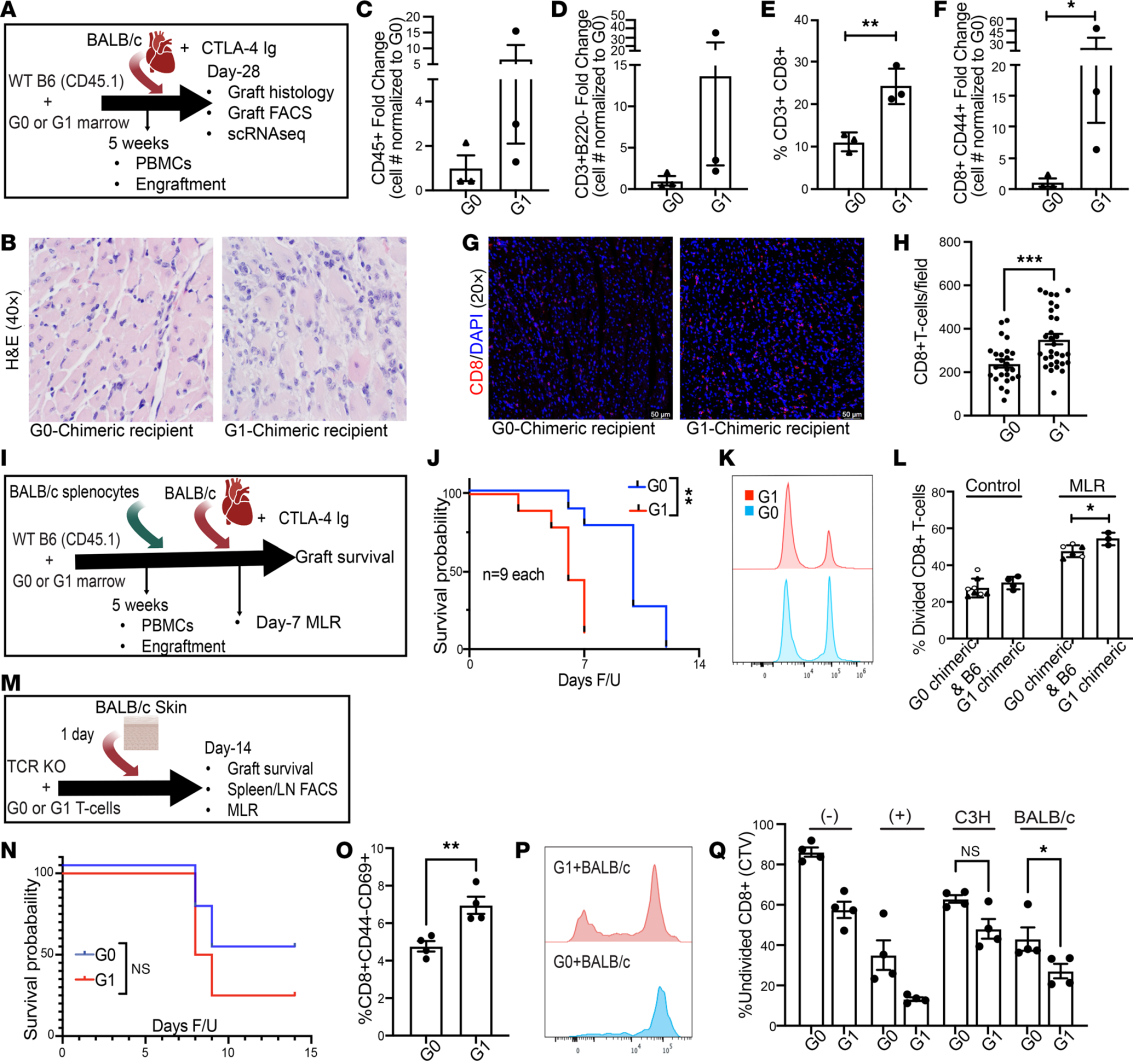
Variant APOL1 BAC-Tg mice demonstrate accelerated allograft rejection in allogenic transplant models. (**A**) Schema of the heterotopic allogenic heart transplant model. G1 or G0 bone marrow (B6/45.2) was transplanted into B6/45.1 syngeneic hosts to create G1- or G0-chimeric mice. BALB/c donor hearts were transplanted into G1- or G0-chimeric recipient mice under costimulation blockade (*n* = 4 each). (**B**) H&E photomicrographs of allografts from G1- versus G0-chimeric recipients. Original magnification, ×40. (**C**–**F**) Flow cytometry of allograft cells for (**C**) CD45^+^CD45.2^+^ cells, (**D**) total T cells (CD3^+^B220^–^), (**E**) CD8^+^ T cells, and (**F**) CD8^+^CD44^+^ T cells (*n* = 3 each). (**G**) Representative IF images and (**H**) quantification (high-power field) of CD8 staining in allografts from G0- and G1-chimeric recipients. Scale bars: 50 μm. (**I**) G0- or G1-chimeric mice were immunized (day 14) with BALB/c splenocytes, followed by BALB/c heart transplantation (*n* = 9 each). (**J**) Allograft K-M survival curves in G1 versus G0-chimeric recipients. (**K**–**L**) Proliferation plot and bar graphs comparing MLRs of CD8^+^ T cells (to BALB/c stimulators) at day 7. Cells were from G1-chimeric, G0-chimeric, and WT-B6 nonchimeric recipients (white circles) of BALB/c hearts (*n* = 3 each). (**M**) Schema of T cell transfer (25 × 10^6^/mouse) from G1 or G0 BAC-Tg mice into TCR-KO recipients (*n* = 4 each). (**N**) K-M curves showing skin graft survival. F/U, follow-up. (**O**) Bar graph quantification of CD8^+^CD44^–^CD69^+^ activated T cells from the same mice. (**P**) Proliferation plot and (**Q**) bars comparing MLRs of CD8^+^ T cells (to BALB/c stimulators) at day 14. Cells were from G1 and G0 TCR-KO recipients. Graphs show the mean ± SEM. *P < 0.05, **P < 0.01, and ***P < 0.001, by Mann-Whitney *U* test; ns = *P* > 0.05. (–), unstimulated; (+), anti-CD3/anti-CD28; C3H, third-party controls.

Previous data suggest a role for APOL1-RVs in kidney allograft rejection (12–14). At baseline, neither G0 nor G1 BAC-Tg CD8^+^ T cells showed evidence of preexisting BALB/c-specific reactivity in a mixed lymphocyte reaction (MLR) (Supplemental Figure 7, A–D). To evaluate the effect of APOL1-RVs expressed on immune cells in an allotransplantation model, we generated bone marrow chimera by transplanting marrow cells from unsensitized G0 and G1 BAC-Tg mice (on a B6/45.2 background) into congenic B6/45.1 hosts. B6/45.2 chimerism was confirmed starting at 5 weeks in B6/45.1 hosts (both H-2^b^) (Supplemental Figure 7E). At 7 weeks, we performed mismatched heterotopic heart transplantations, in which BALB/c (H-2^d^) donor hearts were transplanted into G0- or G1-chimeric mice (*n* = 4 each) with costimulation blockade using CTLA4-Ig (Figure 6A). Since untreated recipients reject mismatched allografts within 7 days (23), we treated recipient mice with a low dose of CTLA4-Ig (costimulatory blockade), which is a widely accepted, clinically relevant model to slow rejection and reduce, but not eliminate, alloreactive T cell immunity (24), thereby facilitating assessments of alloresponses between groups. Mice were monitored for 28 days (see Methods and Supplemental Figure 7F), and at termination, the surviving allografts (*n* = 3 each group) were homogenized and the infiltrate examined by flow cytometry. H&E staining of allografts revealed increased immune infiltrates and tissue necrosis in allografts of G1 recipients (Figure 6B). Flow cytometric analysis identified increased CD45^+^ cells (Figure 6C) and total T cells (Figure 6D) in allografts from G1-chimeric recipients, while CD8^+^ T cells as well as CD8^+^CD44^+^ T cells were both significantly increased (Figure 6, E and F). The increase in CD8^+^ T cells in G1-chimeric recipients was orthogonally confirmed by immunofluorescence in allograft tissue (Figure 6, G and H). CD4^+^ T cells, CD4^+^CD44^+^ T cells, and innate immune cells (CD3^–^B220^–^) also tended to be increased in G1-chimeric recipient hearts (Supplemental Figure 7, G–I).

Next, we evaluated BALB/c-specific CD8^+^ T cell proliferative responses via MLR after in vivo sensitization with BALB/c splenocytes. We found that sensitized G1 BAC-Tg CD8^+^ T cells had increased proliferative responses against BALB/c stimulators compared with G0 cells (Supplemental Figure 7, C and D). On the basis of these data, we immunized G0 and G1 chimeras with BALB/c splenocytes, followed on day 14 by BALB/c heart transplantation with costimulation blockade (*n* = 9 each, sensitized model) (Figure 6I). BALB/c-immunized G1-chimeric recipients showed significantly reduced allograft survival compared with G0-chimeric recipients (Figure 6J). Splenocytes from a subset of immunized and transplanted G1-chimeric mice at post-transplantation day 7 showed higher CD8^+^ and CD4^+^ T cell activation than did G0 chimeras (Supplemental Figure 7, J and K). At day 7, an MLR using BALB/c stimulators revealed increased proliferation of CD8^+^ T cells from G1-chimeric recipient spleens versus G0 or nonirradiated, nonchimeric WT B6/45.1 recipients (Figure 6, K and L). Hence, chimeric G1 BAC-Tg mice demonstrated enhanced CD8^+^ T cell graft infiltration, antigen-specific responsiveness, and accelerated rejection compared with G0-chimeric recipients in the allogenic heart transplant model.

To specifically study APOL1-RV–carrying T cells during alloresponses, we performed transplantation of BALB/c skin grafts into B6-TCRβ–KO (TCR-KO) mice (see Methods; *n* = 4 each). On day 1, we transferred 25 × 10^6^ G1 or G0 T cells into TCR-KO mice (Figure 6M). We used total T cells here, since baseline MLRs against BALB/c were similar between the mouse lines, suggesting an absence of differences in preexisting heterologous T cell memory. In this setup, T cells are provided from G0 or G1 animals, while innate immune and antigen-presenting cells are derived from the TCR-KO host in each case. As expected, skin grafts were highly immunogenic and were rapidly rejected, although G1 T cells tended to reject skin grafts more quickly (Figure 6N). At day 14, splenic and LN evaluation revealed an increase in recently activated CD8^+^ T cells, i.e., CD8^+^CD44^–^CD69^+^ T cells in G1 T cell–recipient animals (Figure 6O and Supplemental Figure 7L). Both G1 CD8^+^ and G1 CD4^+^ T cells showed significantly increased proliferation in MLR experiments with BALB/c stimulator cells versus the respective G0 T cells (Figure 6, P and Q, and Supplemental Figure 7M, respectively). These data support a role for APOL1-RVs in T cells in the promotion of alloreactivity and graft rejection.

### Transcriptome analyses from BAC-Tg CD8^+^ T cells reveals a role for enhanced calcium-mediated calcineurin/NFAT signaling in variant mouse T cell activation and expansion.

**Figure 7:**
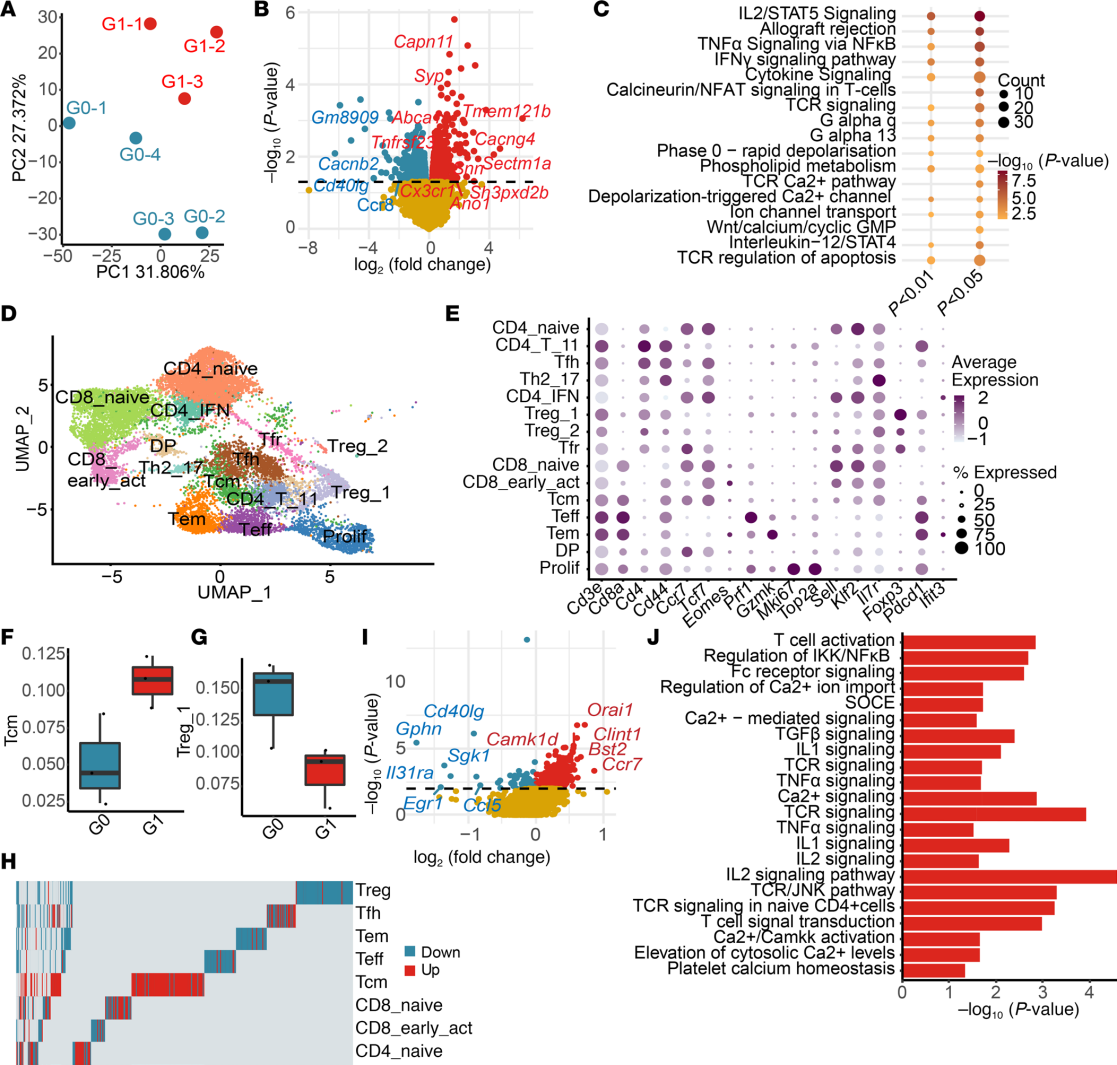
Transcriptome analyses reveal enhanced calcium-mediated calcineurin/NFAT signaling in G1 CD8^+^ T cells. (**A**) Principal component (PC) analysis of the CD8^+^ T cell transcriptome of G1 versus G0 BAC-Tg mice. (**B**) Volcano plot of significant DEGs in G1 versus G0 transcriptomes. Dashed line *P* = 0.05; selected Ca^2+^ signaling–related transcripts are highlighted. (**C**) Functional enrichment analyses of significant DEGs (at *P* < 0.01 and *P* < 0.05, respectively). (**D**) Uniform manifold approximation and projection (UMAP) of scRNA-seq of CD3^+^ T cells from G1 and G0 recipient allografts (*n* = 3 each). (**E**) Bubble plot shows expression of canonical T cell markers for subset annotation. Box plots show proportions of infiltrating (**F**) Tcms and (**G**) Tregs in each sample (median/IQR; whiskers represent ± 1.5 box heights). (**H**) Significant DEGs (*P* < 0.01) within each T cell subset in G1 versus G0 comparisons. (Each column represents 1 gene; red = upregulated, blue = downregulated.) (**I**) Volcano plot of the DEGs from infiltrating Tcms in G1 versus G0 comparisons (dashed line *P* = 0.01). (**J**) Functional enrichment analyses with the top 50 DEGs (ranked after downsampling) in Tcms. CD8_early_act, activated CD8^+^ T cells in the early phase; DP, CD4^+^/CD8^+^ double-positive; Prolif, proliferating cells.

To investigate the mechanism of APOL1-RVs in T cells, we performed RNA-seq of ex vivo–stimulated naive CD8^+^ T cells from G1 and G0 mice (*n* = 3 vs. 4, respectively; see Methods). Batch-normalized principal component analyses of transcriptome-wide profiles showed clustering of CD8^+^ T cells by genotype (Figure 7A). Significantly differentially expressed genes (DEGs) (445 upregulated and 300 downregulated; limma *P* < 0.05; Figure 7B and Supplemental Table 5) were identified and ranked for enrichment analyses. *APOL1* transcripts were increased in the G1 T cell transcriptome despite similar BAC copies, consistent with the quantitative PCR (qPCR) results (Figure 3A vs. Supplemental Figure 8A). The top enriched pathways at different *P* value thresholds are plotted in Figure 7C. Canonical immune response pathways related to TCR stimulation were identified as significantly enriched in G1 CD8^+^ T cells, including IL-2/STAT5, NF-κB, and calcineurin/NFAT signaling pathways. Interestingly, multiple pathways related to calcium transport/calcium signaling (Figure 7C and Supplemental Figure 8, B and C) were identified as enriched. Selected dysregulated genes representing calcium channels or calcium-activated signals are labeled in Figure 7B.

To study G1 CD8^+^ T cell subsets, we evaluated the single-cell transcriptome of graft-infiltrating T cells from the BALB/c cardiac allografts harvested from chimeric G1 or G0 recipient mice at 4 weeks (*n* = 3 each; Figure 6A). We flow-sorted live CD3^+^ cells from allografts and performed single-cell RNA-seq (scRNA-seq) using a hashtagging approach (25) to multiplex the 6 samples during sequencing (*n* ~17,000 total cells; Supplemental Figure 8, D and E). As also seen in Figure 6E, deconvolution of hashtags revealed that markedly more T cells had infiltrated allografts in G1 recipients (Supplemental Figure 8F). T cell subsets were annotated as described before (26), and 22 clusters were identified (Figure 7, D and E). Total numbers of all CD8^+^ T cell subsets were markedly increased in G1-chimeric recipients (Supplemental Figure 8F). G1 sample 5 was an outlier in these analyses, with high numbers of naive CD4^+^ and CD8^+^ T cells. Hence, we evaluated the proportions of infiltrating cells within each graft after excluding naive T cells (Supplemental Figure 8G). We observed that the proportions of Tcms, T effector cells (Teffs), and T effector/memory cells (Tems) were all increased in G1-chimeric recipients whereas proliferating and Treg proportions were reduced in G1- versus G0-chimeric recipients (Figure 7, F and G, and Supplemental Figure 8H).

We then evaluated DEGs within these annotated cell clusters. Given the imbalance in the number of infiltrating cells between G1 and G0 recipients (Supplemental Figure 8F and Supplemental Figure 9A), we performed random downsampling (bootstrapping). This approach equalized the number of cells analyzed in each iteration between G0 and G1recipients, minimizing the potential for bias during DEG identification. Within each cell type, significant DEGs were identified from every bootstrapped test and ranked by the frequency of their occurrence. DEGs identified from 50% of bootstrapped samples were considered robust DEGs and then ranked by their mean fold change. Supplemental Table 6 lists the top-ranked DEGs (by average fold change) in each cell type. Enrichment analyses of DEGs in Teffs, Tems, naive CD8^+^ T cells, and proliferating T cells using the downsampling approach are shown in Supplemental Figure 9B. G1 naive CD8^+^ T cells showed enrichment of canonical TCR signaling and IL-2/STAT5 and TNF-α/NF-κB signaling pathways, suggesting basal activation (Supplemental Figure 9B). Tcms showed the greatest transcriptional perturbation by DEG number (*n* = 642; Figure 7, H and I). In Tcms, the top 50 significant DEGs (by fold change) were subjected to enrichment analyses (Figure 7J). Consistent with the bulk sequencing data, the calcium/calmodulin/kinase axis, TCR activation, calcineurin/NFAT signaling, and calcium and TNF-α/NF-κB signaling were significantly enriched in G1 Tcms. Interestingly, the top-ranked upregulated DEG in G1 Tcms was *Ccr7*, consistent with flow data for G1 BAC-Tg mice. ORAI1, a calcium channel activated by STIM1 with a key role in store-operated calcium entry (SOCE), was highly upregulated in G1 Tcms (Figure 7I). For sensitivity analyses, we excluded the outlier G1 sample 5 and repeated the downsampling approach to reidentify significant DEGs. DEGs from this sensitivity analyses demonstrated significant overlap with originally identified DEGs within each cell type including Tcms, naive CD8^+^ T cells, and naive CD4^+^ T cells (hypergeometric *P* < 0.001; Supplemental Figure 9C). Similarly, DEGs identified with or without the downsampling approach also showed significant overlap (Supplemental Figure 9C), confirming our results.

### Role of ER calcium depletion and SOCE in T cell activation by the variant APOL1.

Our unbiased transcriptomics relayed enrichment of calcium (Ca^2+^) signaling pathways in G1 CD8^+^ T cells. APOL1 is a monovalent cation channel partly localized in the plasma membrane (8), and it was recently reported that increased channel activity in APOL1-RVs at the cell surface (vs. G0 APOL1) initiates a mechanism for cytotoxicity in epithelial cells by altering the resting membrane potential (9, 10). Here, plasma membrane depolarization led to phospholipase C activation via GPCRs with IP3-mediated Ca^2+^ channel activation (pathways also enriched in G1 CD8^+^ T cells; Figure 7, C–J), causing ER-Ca^2+^ leak and depletion (8). In T cells, TCR activation impinged on IP3-mediated Ca^2+^ release and ER-Ca^2+^ depletion, in turn stimulating SOCE (via calcium release–activated calcium [CRAC] Orai-Stim1 channels) and calcineurin activation. Indeed, *Orai1*, a key participant in SOCE, was highly upregulated in G1 Tcms. As reported in epithelial cells, we also confirmed that APOL1 expression occurred on the plasma membrane of BAC-Tg (Supplemental Figure 10A) and human (Supplemental Figure 10, B–E) CD8^+^ T cells.

**Figure 8:**
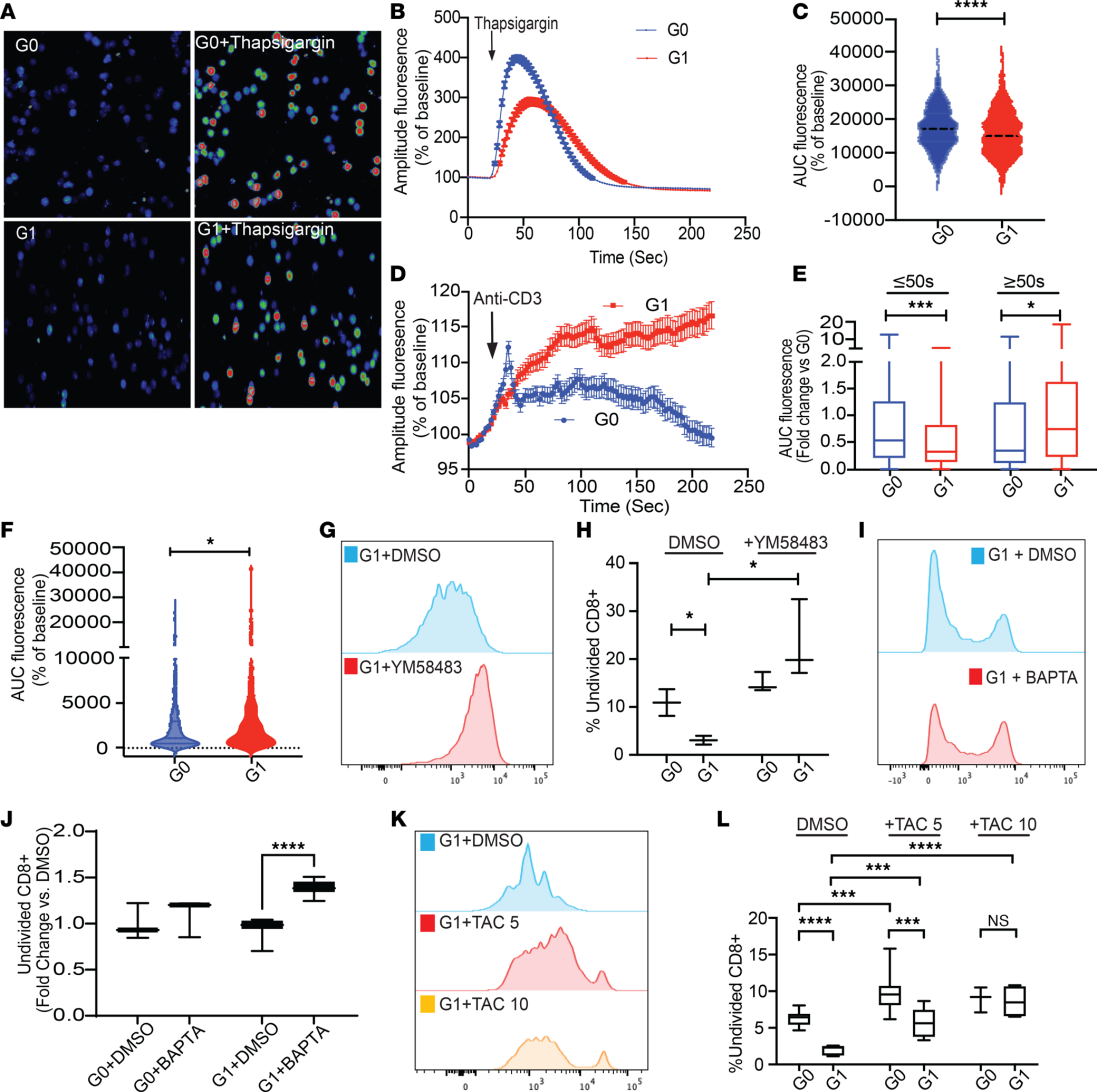
ER calcium depletion causes activation of variant APOL1 CD8^+^ T cells. (**A**–**C**) (**A**) Representative images (dots = T cells), (**B**) mean calcium flux curves, and (**C**) corresponding average AUCs of fluorescence measured from live G0 and G1 CD8^+^ T cells in response to thapsigargin (*n* = 5 mice each). Original magnification, ×20 (**A**). (**D**–**F**) Summarized calcium flux curve (**D**), AUCs of early versus late fluorescence (<50 seconds or >50 seconds) (**E**), and total fluorescence from CD8^+^ T cells from G1 versus G0 after anti-CD3-stimulation (>500 cells, *n* >6 mice) (**F**). (**G**) Representative plot and (**H**) quantified undivided total CD8^+^ T cells from G1 versus G0 mice with YM-58483 at 50 nM. (**I**) Proliferation plots of G1CD8^+^ T cells treated with BAPTA-AM (0.3 μM) and DMSO and (**J**) quantification of proliferation of G0 or G1 CD8^+^ T cells treated with BAPTA-AM (0.3 μM) or DMSO (triplicates of 5 mice), respectively. (**K**) Representative plots of G1CD8^+^ T cells treated with TAC at 5 and 10 ng/mL and (**L**) and the corresponding quantification of proliferation of G0 and G1 CD8^+^ T cells with 0, 5, and 10 ng/mL TAC. Box-and-whisker plots show the median and range of triplicates pooled from more than 5 mice each. *P < 0.05, ***P < 0.001, and ****P < 0.0001, by 1-way ANOVA with Tukey’s post hoc test.

To first investigate ER-Ca^2+^ stores, CD8^+^ T cells from G0 and G1 mice were stimulated ex vivo for 48 hours (anti-CD3–coated plates for adhesion; *n* = 5 mice each). ER-Ca^2+^ stores reflect a balance of Ca^2+^ entering the ER via sarco/endoplasmic reticulum calcium ATPase (SERCA) pumps, and Ca^2+^ release via the IP3R and ryanodine receptors (27). We assayed changes in cytosolic Ca^2+^ concentration ([Ca^2+^]_cyt_), which reflects efflux from ER-Ca^2+^ stores, by live cell imaging using Fluo-4–AM dye immediately after the addition of thapsigargin (a SERCA inhibitor) and simultaneously precluded ECF-Ca^2+^ entry using EGTA. Here, both amplitude (Supplemental Figure 10F) and AUC (Figure 8, A–C) of the thapsigargin-induced [Ca^2+^]_cyt_ peak were significantly reduced in G1 CD8^+^ T cells, confirming ER-Ca^2+^ depletion in G1 versus G0 CD8^+^ T cells.

We surmised that ER-Ca^2+^ depletion in G1 CD8^+^ T cells would promote earlier engagement of SOCE during TCR stimulation. To study Ca^2+^ flux following TCR stimulation, CD8^+^ T cells from G0 and G1 mice (*n* = 6 each) were stimulated (as above), and [Ca^2+^]_cyt_ was assayed after fresh replenishment of anti-CD3. An average [Ca^2+^]_cyt_ curve summarizing all G1 and G0 CD8^+^ T cells is shown in Figure 8D. G1 CD8^+^ T cells demonstrated a blunted early increase in [Ca^2+^]_cyt_ with a significantly decreased amplitude and AUC of the early peak (<50 seconds), indicating a reduced ER-Ca^2+^ release versus G0 (Supplemental Figure 10G and Figure 8E, respectively). However, delayed [Ca^2+^]_cyt_ release (>50 seconds after anti-CD3), as well as overall [Ca^2+^]_cyt_ — expressed as the AUC — were significantly higher in G1 CD8^+^ T cells versus G0 (Figure 8, D–F). These data confirm ER-Ca^2+^ depletion in G1 APOL1 CD8^+^ T cells with a blunted early rise in [Ca^2+^]_cyt,_ that was followed by sustained [Ca^2+^]_cyt_ in later phases of TCR stimulation.

SOCE, mediated by the ORAI1-STIM1 complex, replenishes ER-Ca^2+^, contributing to the later part of the [Ca^2+^]_cyt_ curve and promoting T cell activation via calcineurin/NFAT. Consistent with the role played by SOCE specifically in G1 CD8^+^ T cells, reduced the doses of the CRAC channel inhibitor YM-58483 (50 nM; IC_50_ = 330 nM) (28) obviated the increased proliferation and the reduced viability seen in TCR-stimulated G1 CD8^+^ T cells versus G0 CD8^+^ T cells (Figure 8, G and H, and Supplemental Figure 10H).

To further test the role played by [Ca^2+^]_cyt_ levels, we stimulated G1 and G0 CD8^+^ T cells in the presence of the Ca^2+^ chelator BAPTA-AM (29). At higher concentrations (3 μM, 48 hours), BAPTA-AM promoted cell death; we therefore used 10-fold-reduced concentrations of BAPTA-AM (0.3 μM). We found that G1 CD8^+^ T cells were more sensitive to BAPTA-AM with inhibited proliferation compared with G0 cells (Figure 8, I and J). The calcineurin/NFAT pathway was enriched in G1 CD8^+^ T cells (Figure 7C), and we reasoned that greater activation of this pathway by SOCE would render G1 cells more refractory to inhibition by calcineurin inhibitors (CNIs). We therefore tested CD8^+^ T cell activation in the presence of increasing concentrations of the CNI tacrolimus (TAC) (at 0, 5, or 10 ng/mL). G1 CD8^+^ T cells showed greater proliferation when compared with G0 cells at 0 and 5 ng/mL, but differences in proliferation were abolished only at 10 ng/mL (Figure 8, K and L). Similarly, Tcm expansion was dose dependently decreased in G1 CD8^+^ T cells at 0, 5, and 10 ng/mL but not obviated (vs. G0) at 10 ng/mL (Supplemental Figure 10I).

### APOL1-RV kidney transplant recipients show increased rejection with higher TAC trough levels.

Our studies above suggested that APOL1 G1 variant CD8^+^ T cells sustained ER-Ca^2+^ leak and depletion (8), followed by increased SOCE, calcineurin/NFAT signaling, and resistance to TAC inhibition, promoting Tcm expansion and rejection. To evaluate this in human kidney transplantation, we used samples from the Clinical Trials in Organ Transplantation-19 (CTOT-19) study. From this randomized trial funded by the National Institute of Allergy and Infectious Diseases (NIAID), NIH (30), which tested the effects of early TNF-α blockade on graft outcomes (*n* = 225), we obtained DNA from kidney recipients and resolved APOL1 genotypes (see Methods) (12, 31). Among 210 of 225 CTOT-19 kidney recipients with genotype data, 65 (30.9%) had APOL1-RV genotypes (49 of 65 had the G1 genotype); 81 (38.57%) were AAs, and 73% of the AAs had APOL1-RV genotype (Table 2).

**Figure 9:**
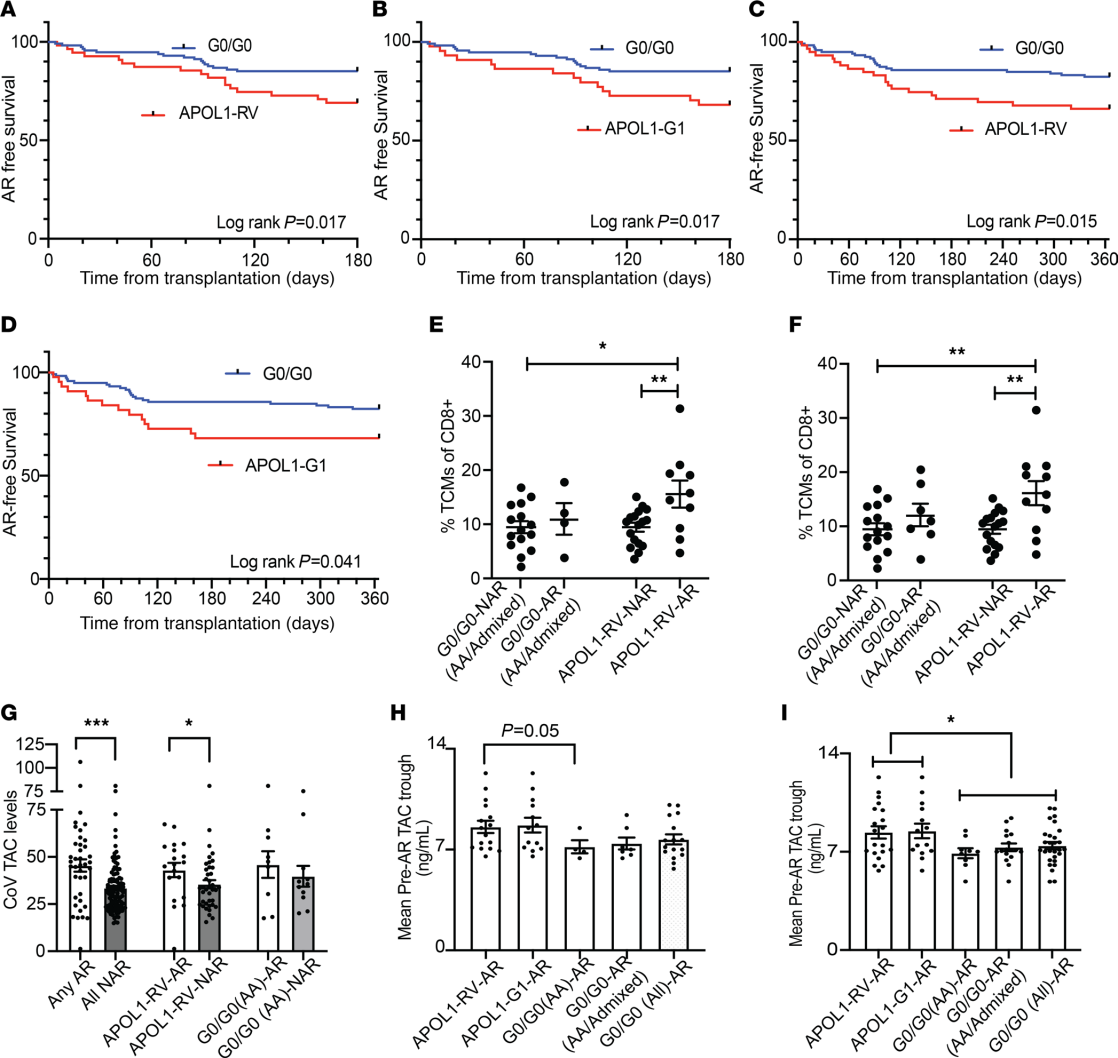
Tcm expansion increases the risk of rejection in APOL1-RV kidney transplant recipients with higher TAC trough levels. The CTOT-19 cohort was used to validate our findings. (**A**–**D**) K-M curves show time-to-event analysis of (**A**) AR by 6 months in any APOL1-RV, (**B**) in APOL1-G1 recipients, and corresponding data for (**C** and **D**) AR by 12 months, respectively, versus G0/G0. (**E** and **F**) Dot plots of the percentage of Tcms (CD8^+^CD45RO^+^CD28^+^CD27^+^ among T cells) before transplantation by APOL1 genotype (**E**) in AA/admixed patients with or without AR by 6 months and (**F**) in AA/admixed patients with or without AR by 24 months. (**G**) CoV of pre-AR TAC troughs between AR and NAR, by APOL1 genotype. (**H** and **I**) Bar graphs show mean pre-rejection TAC troughs by APOL1 genotype of (**H**) 6-month AR versus NAR and (**I**) any time AR versus NAR. Graphs show the mean ± SEM. *P < 0.05, **P < 0.01, and ***P < 0.001, by Mann-Whitney *U* test.

Rejection diagnoses were obtained from both local and central laboratory reports (Supplemental Table 7 and Methods) (30). We included subclinical and clinical ABMR and TCMR (including borderline) diagnoses made by central or local pathology and refer to them as “AR.” Among 210 patients, 34 developed AR by 6 months, 42 by 12 months, and 49 by 24 months (end of study). Within 6, 12, and 24 months, patients with at least 1 biopsy who did not have the AR diagnosis were classified as “no rejection” or “NAR.” We found that kidney recipients with the APOL1-RV genotype had a significantly increased incidence of AR by 6 months (Table 3). In time-to-event analyses, any APOL1-RV or APOL1-G1 genotype was associated with a significantly increased risk of AR by 6 and 12 months (Figure 9, A–D, respectively). The association between APOL1-RV copies and 6- or 12-month AR was identifiable in additive models (Supplemental Figure 11, A and B) (12, 14). In bivariate Cox models for time to 6-month rejection, adjusted for the recipient’s age, CMV status (i.e., covariates significantly different between APOL1-RV vs. G0/G0 in Table 3), or randomization limb, APOL1-RV remained significantly associated with AR. Adjustment for genetic ancestry attenuated the significance of association of APOL1-RV with AR, albeit with similar HRs (Supplemental Table 8). Inclusion of AR events up to 24 months tended to attenuate the significance of association, suggesting an association of APOL1-RVs with early AR (Supplemental Figure 11C).

Since the CTOT-19 cohort had only 2-year follow-up with few graft losses (30), we evaluated the published association of APOL1-RVs in recipients using a meta-analysis approach. We included studies that utilized (a) the APOL1-RV genotype of the recipients, (b) adjustment for the genetic ancestry of the recipients, and (c) additive modeling for APOL1-RV risk computation (in place of recessive models). Of the 4 published studies that tested associations of graft survival with recipient APOL-RV status (12–14, 32), only 2 adjusted for recipient genetic ancestry with additive modeling. Together, these studies included a sample size of 2,281 recipients from 4 independent cohorts. In these meta-analyses, the presence of each APOL1-RV copy associated with an increased hazard of death-censored graft loss in Cox models adjusted for genetic ancestry, as well as clinical covariates included in each cohort (Supplemental Figure 11D).

We next investigated the CD8^+^ T cell phenotype of individuals with APOL1-RVs who developed early AR (by 6 months; *n* = 17) and compared these with APOL1-RV NARs of AA/admixed ancestry (*n* = 39) as well as G0/G0 AA/admixed ancestry with AR (*n* = 7), or with NARs (*n* = 36; total 6-month AR data from *n* = 99) (Table 3). From flow data collected at multiple time points, we focused on the pre-transplantation visit (visit 0), i.e., before initiation of immunosuppression or infliximab (visit 0 flow data available for 56 of 99 patients). Within this dataset, we compared the pre-transplant CD8^+^ T cell phenotype of patients with or without AR by 6 months. We observed that patients with APOL1-RV ARs had significantly higher proportions of Tcms (CD8^+^CD45RO^+^CD28^+^CD27^+^) than did APOL1-RV NARs or G0/G0 NARs (Figure 9E and Supplemental Figure 11E), consistent with the murine data. Other CD8^+^ T cell subsets including Temra (CD8^+^CD45RA^+^CD28^–^CD27^–^), Teff (CD8^+^CD45RO^+^CD27^–^), and naive CD8^+^ (CD8^+^CD45RA^+^CD28^+^CD27^+^) T cell fractions were not significantly different between any of the groups (Supplemental Figure 11, F–H). The proportion of Tcms before transplantation was still significantly higher in APOL1-RV ARs when all AR episodes during the study were collated from APOL1-RV and G0/G0 AA/admixed individuals (Figure 9F).

**Figure 10:**
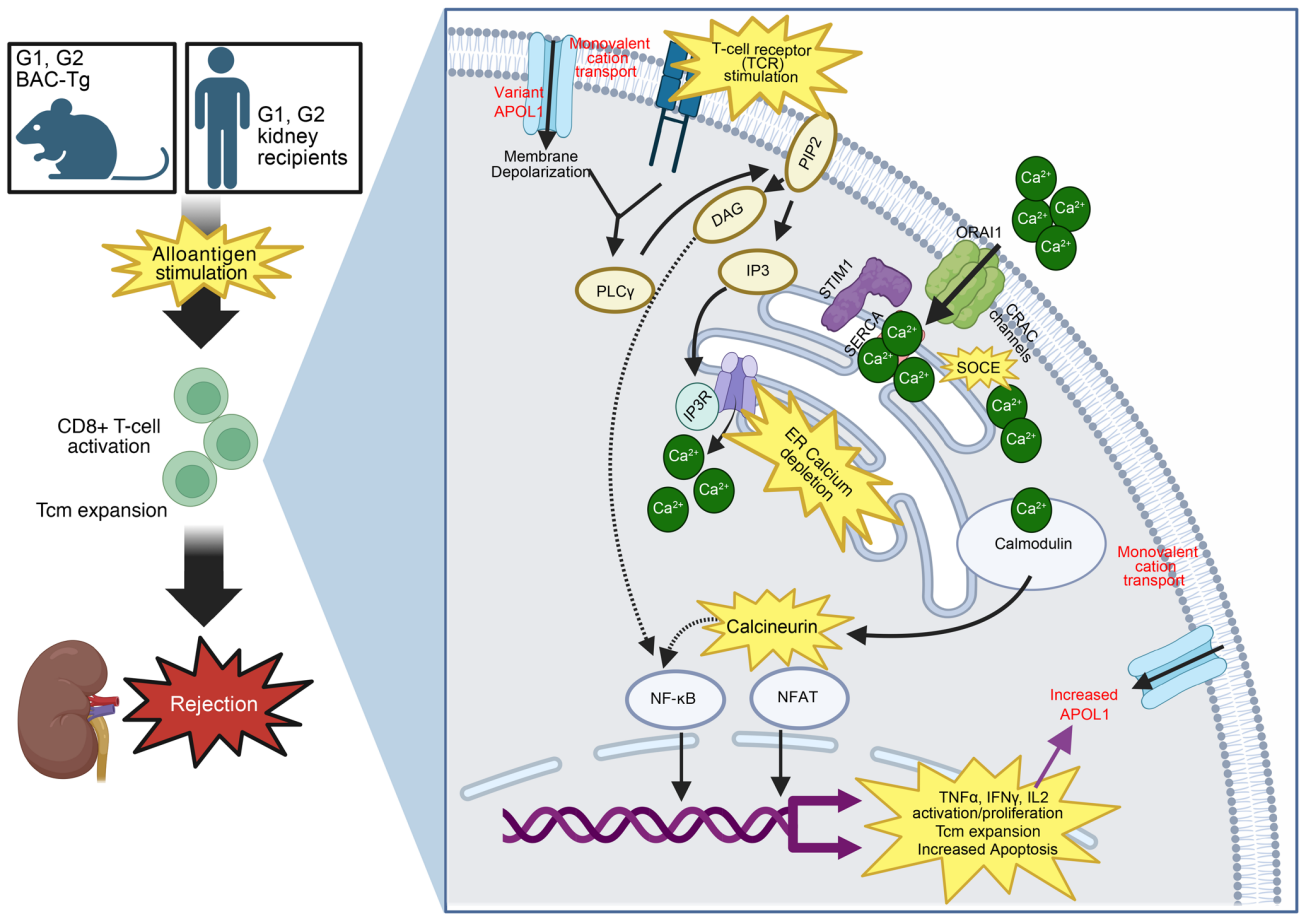
Proposed schema of mechanism by which APOL1 risk variants in T cells promote allograft rejection. Expression of APOL1-RVs (a cation transporter) in BAC-Tg CD8^+^ T cells causes membrane depolarization and activates PLCγ-PIP2-IP3, driving sustained ER-Ca^2+^ leak via IP_3-_channels. Following TCR ligation, preexisting ER-Ca^2+^ depletion leads to sustained SOCE via STIM1-ORAI1. Downstream Ca^2+^-calmodulin-calcineurin activates NFAT and NF-κB pathways, and enhanced cytokines, activation, and Tcm expansion, accompanied by activation-induced cell death. Clinically, recipients with APOL1-RVs developed increased AR when they had elevated Tcms before transplantation and despite significantly higher TAC levels versus G0-AA-AR. Figure created with BioRender.com.

To investigate TAC dose dependency, we utilized mean TAC trough levels collected throughout the study. Overall, ARs had higher coefficients of variation (CoV) of TAC troughs versus NARs, suggesting TAC variability and nonadherence as a predisposition for AR (Figure 9G). This finding was consistent among APOL1-RVs. Interestingly, APOL1-RV ARs had higher TAC trough levels before AR (i.e., pre-rejection TAC troughs) before 6-month AR (Figure 9H; *P* = 0.05), and before any AR (Figure 9I) compared with APOL1-G0/G0 ARs (including both AAs/admixed and the entire cohort) and suggest that AR occurred in APOL1-RV recipients despite higher TAC trough levels. Together, these data validate the increased risk of AR with APOL1-RVs and support the CNI-mediated T cell activation, Tcm expansion, and allograft injury seen in our murine studies (summarized in Figure 10).

## Discussion

APOL1 FSGS is consistent with a podocyte-centric paradigm of APOL1-RV toxicity, and data from kidney transplantation support increased DCAL in APOL1-RV donor kidneys compared with G0/G0 donor kidneys. A growing body of clinical data has now also reported an association with APOL1-RVs in kidney recipients with TCMR and DCAL (12–14). However, epidemiologic reports cannot fully unravel the association of APOL1-RVs with rejection, since AA ancestry, which is highly linked with APOL1-RV carriage (14, 33), is also associated with a higher risk of rejection according to clinical factors, pharmacogenomic loci influencing drug metabolism (15), and socioeconomic indicators (14, 33). Thus, examination of the specific role and mechanisms of APOL1-RVs during kidney rejection is important since an APOL1 inhibitor is already in phase III human trials (34) and will have therapeutic implications. In the current series of experiments, we used a BAC-Tg mouse model to investigate APOL1-RVs in T cell responses leading to TCMR and separated this from associations with AA ancestry. We also performed parallel key validation studies using transplanted and nontransplanted patient samples.

We identified a clear CD8^+^ T cell phenotype in adult G1 and G2 BAC-Tg mice versus G0 mice, with expansion of Tcms promoting inflammatory responses and allorejection. While we focused on backcrossed G1 mice for phenotyping experiments, we performed selected validation in G2 hybrid BAC-Tg mice. The differential phenotype with APOL1-RVs was observed in ex vivo–stimulated naive, as well as antigen-experienced, CD8^+^ T cells, and G1 T cell responses were ameliorated by an APOL1 antagonist. We observed increased alloantigen-specific responses against mismatched heart transplants with G1 bone marrow chimeras and in MLR studies with isolated T cell transfers. Taken together, our work demonstrates an excitatory role for APOL1-RVs in T cells, and identifies a potentially targetable phenotype in transplantation, given the availability of APOL1 inhibitors.

Unbiased transcriptome analyses suggested altered Ca^2+^ signaling as an important underpinning mechanism in APOL1-RV CD8^+^ T cells. ER-Ca^2+^ depletion has been previously implicated as a mechanism of cytotoxicity of APOL1-RVs in epithelial cells. In this model, plasma membrane depolarization by surface APOL1-RVs led to phospholipase C activation via GPCRs with persistent IP3-Ca^2+^ channel activation and ER-Ca^2+^ depletion (8). We experimentally confirmed surface expression of APOL1-RVs in CD8^+^ T cells and demonstrated depleted ER-Ca^2+^ stores in G1 CD8^+^ T cells. Furthermore, phospholipase C and GPCR signaling were also consistently identified in G1 CD8^+^ T cell transcriptomes as dysregulated pathways supporting the involvement of this axis. Hence, our findings from G1 CD8^+^ T cells paralleled epithelial cells (8), with surface APOL1-RVs causing secondary ER-Ca^2+^ depletion. However, unlike in epithelial cells, in T cells, IP3-mediated ER-Ca^2+^ depletion drives SOCE-mediated [Ca^2+^]_cyt_ levels, which is central to calcineurin/NFAT activation after TCR stimulation. In line with this concept, we observed sustained increases in [Ca^2+^]_cyt_ levels with TCR ligation in G1 CD8^+^ T cells, and perturbations of [Ca^2+^]_cyt_ with TCR stimulation disproportionately affected G1 CD8^+^ T cells. Inhibition of G1 CD8^+^ T cell proliferation required higher TAC levels, corresponding to increased calcineurin/NFAT activation. The increase in SOCE that we observed in G1 CD8^+^ T cells could also explain the Tcm expansion, as SOCE has been shown to be critical for upregulation of genes involved in oxidative phosphorylation, which promotes metabolic reprogramming and memory formation (35). Upregulated TNF-α in G1 CD8^+^ T cells could also be related to increased Ca^2+^-dependent or -independent NF-κB activation downstream of the TCR (36).

Our study has important implications. Our translational data suggest that recipients with APOL1-RVs may need higher levels of TAC to achieve sufficient immunosuppression. Notably, we observed that even at TAC levels of approximately 10 ng/mL, there was incomplete inhibition of Tcm expansion during TCR stimulation in G1 CD8^+^ T cells. Moreover, higher levels of TAC increase the risk of infection and nephrotoxicity and may not be clinically feasible. A recent clinical alternative to TAC is costimulation blockade using belatacept (37). We note that sensitized G1 BAC-Tg mice rejected heart allografts despite costimulation blockade with CTLA4-Ig. However, whether our experimental findings have clinical implications for belatacept-based maintenance of immunosuppression will require future clinical studies. Since agents targeting APOL1-RVs are also approaching clinical trials (34), we believe our data have a number of therapeutic implications.

Furthermore, our data raise the possibility that individuals with APOL1-RVs who have a greater proportion of Tcms prior to transplantation may be at highest at risk for early AR, and Tcm frequency measurement may allow risk stratification for individualized monitoring. However, further studies are needed to fully understand how Tcm expansion modifies the rejection risk for individuals with APOL1-RVs. A similar role for recipient APOL1 in nonkidney solid organ rejection also needs future evaluation. While the data presented here as well as in other recently published studies suggest a role for recipient APOL1 genotype in kidney transplant outcomes, it is not yet clear whether the combination of recipient-plus-donor APOL1-RVs presents a higher risk of graft loss than either one alone. Larger datasets with enrichment of data on AA donors and recipients with the APOL1-RV genotype, like the data from the APOLLO consortium, will help clarify this question (38). Since viral insults, immune stimulation, and IFN-γ excess (7) all stereotypically induce APOL1 FSGS in native kidneys, and we observed a reduced risk for some microbial diseases in combination with proinflammatory cytokine excess with APOL1-RVs from human and animal data, a potential upstream role for T cell activation in APOL1-mediated FSGS also needs investigation.

Our study has some limitations. Haplotypic differences may influence APOL1-RV toxicity (39). All 3 BAC clones used here contain a lysine (K150) that is found only in the G0 (reference) haplotype, and these clones are not shared with the haplotype associated with APOL1-RVs in AA individuals. However, we support our data with analogous findings in human cohorts representing the correct AA haplotype. Next, a role for APOL1-RVs during thymus development influencing the adult T cell phenotype was not addressed. APOL1 is expressed in many immune cells including activated T cells (12), and recent data report a proinflammatory role for APOL1-RVs in macrophages (40). While T cell transfer experiments in TCR-KO mice showed increased BALB/c-specific alloresponses in G1 BAC-Tg T cells (without accompanying G1 innate immune cells), in the context of APOL1-RV recipients, alloresponses are likely augmented by proinflammatory APOL1-RV innate immune cells. In our retrospective data on TAC levels from CTOT-19, we also cannot completely rule out the possibility that the higher TAC levels seen in patients with APOL1-RVs could have been influenced by the clinicians’ treatment decisions for these particular patients. While we show an important role for ER-Ca^2+^ depletion and calcineurin activation in G1 T cells, Tcm expansion was not completely inhibited with a CNI. This finding suggests a role for other signaling pathways downstream of APOL1-RVs, such as T cell proliferation and cytokine excess signaling pathways, which need future investigation.

In conclusion, we demonstrate for the first time to our knowledge that APOL1-RVs in T cells induce CD8^+^ T cell activation by promoting ER-Ca^2+^ leak, impinging on SOCE and canonical TCR signaling. Our work demonstrates a causal role for these variants in promoting AR risk after kidney transplantation and provides opportunities for precision therapeutics directed at carriers of APOL1 exonic variants.

## Methods

See Supplemental Methods for details.

### Sex as a biological variable

Animals of both sexes were included, and similar findings were reported in this study.

### BAC-transgenic mouse model

The reference APOL1-BAC-Tg G0 construct was subcloned, and G1 and G2 BACs were generated by site-directed mutagenesis. BAC-Tg mice were generated by pronuclear injection at the Yale O’Brien Center Transgenic Core following a method described by Wang et al. (41). Founders with 2 copies of APOL1-BACs were backcrossed with mice on the C57BL/6J background. For genotyping, the mouse APOL1 copy number was determined using cellular DNA via the ΔΔCt method and compared against human homozygotes. *Rps18* was used as an endogenous control for mouse samples, while *B2M* was used for human samples.

### Animal studies

#### Ex vivo mouse studies.

Whole and naive CD4^+^ and CD8^+^ T cells were isolated from processed mouse tissues (EasySep kits), stained with CellTrace Violet (CTV), and proliferated with anti-CD3/anti-CD28. The effects of an APOL1 inhibitor (MZ-302) on T cell proliferation and cytokine production and the effects of BAPTA-AM (at 0.3 and 3.0 μM), YM-58483 (at 50 nM), or TAC (at 5 and 10 ng/mL) on T cell proliferation were examined. Intracellular cytokine production was assayed (Cytofix/Cytoperm Plus kit with GolgiPlug, no. 555028) after PMA/ionomycin.

#### Flow cytometric analysis.

The harvested cells were first stained with Zombi NIR and the surface markers and followed by fixation/permeabilization using FOXP3/Transcription Factor Staining Buffer (Invitrogen, Thermo Fisher Scientific, 00-5523-00) for intracellular markers. Details on tissue processing, T cell isolation, proliferation, staining, and the published antibodies, fluorochromes, and gating strategies used in this study are provided in Supplemental Methods (42, 43). All samples for flow cytometry were run on a BD Symphony A5 Cell Analyzer.

#### Thapsigargin-mediated cytosolic calcium assay.

CD8^+^ T cells were stimulated on coated 96-well plates for 48 hours. To estimate the total ER Ca^2+^-content, T cells were incubated with Fluo-4 AM and washed with Ca^2+^- and phenol red–free RPMI media containing 2% FBS and 1 mM EGTA. Cells were then stimulated with 10 μM thapsigargin (Invitrogen, Thermo Fisher Scientific, T7459).

#### Anti-CD3–mediated cytosolic calcium assay.

For intracellular Ca^2+^ chelation, BAPTA-AM was loaded alongside Fluo-4 AM. Fluorescence at baseline (20 seconds) and with anti-CD3 (5 μg/mL for 3.5 minutes) was quantified. Increases in [Ca^2+^]_cyt_ were expressed as a percentage of increase in Fluo-4 fluorescence intensity (normalized to baseline).

### In vivo mouse studies

Mice were injected retro-orbitally with 200 μg poly(I:C) (44). Sera were collected at days 1 and 7 after injection. For immunization, splenocytes were isolated from approximately 10-week-old male BALB/c mice and injected intraperitoneally into BAC-Tg animals (~5 × 10^6^/mouse).

#### Murine cytokine assay.

Sera from poly(I:C)-treated mice and supernatants of ex vivo–stimulated CD8^+^ T cells were assessed using the Mouse Cytokine Discovery Assay (Eve Technologies).

### In vitro studies

HEK293T cells were cotransfected with APOL1 plasmid constructs (VA- and VC-, for G0, G1, or G2 constructs from Waldemar Popik, Meharry Medical College) (45), with packaging plasmids to generate mammalian VSV-pseudotyped lentiviral expression constructs (46).

#### Cytotoxicity assays.

PI/annexin V and WST-1 staining was performed using the Pacific Blue Annexin-V Kit with PI (BioLegend, catalog 640928) and the Cell Proliferation Assay Kit (MilliporeSigma, catalog 2210), respectively.

#### Immunoblotting.

HEK293T cells and mouse cells were processed to quantify APOL1 (see Supplemental Methods).

#### qPCR.

Primer sets were designed for all assayed genes via Primer-BLAST (NCBI) (Supplemental Table 9). Gene expression was assayed by reverse transcription quantitative PCR (RT-qPCR) (Applied Biosystems 7500) via the ΔΔCt method and using GAPDH as an endogenous control.

### Transplantation experiments

#### Bone marrow chimera.

Bone marrow cells were prepared from G0 and G1 animals (10–12 weeks old) by mechanically flushing and RBC lysis. Recipient animals received whole-body irradiation (900 cGy) in a gamma irradiator followed by intravenous injection of 5 × 10^6^ bone marrow cells. Donor chimerism was confirmed using congenic markers.

#### Heterotopic heart transplants.

Heterotopic heart transplant experiments were performed as reported before (47, 48). Recipients were treated with CTLA-4 Ig (Abatacept, 10 mg/kg i.p. ~250 mg on day 2). Mixed lymphocyte reactions were performed using irradiated donor splenocytes. Recipient cells were stained with CTV (Invitrogen, Thermo Fisher Scientific), and then donor (stained with CFSE) and recipient cells were cocultured in 24-well plates for 96 hours at 1 × 10^6^ and 1 × 10^5^ cells/well. Positive controls were mouse T cell activator beads (at a bead/cell ratio of 1:1) and negative controls were without stimulation.

#### CD8 immunofluorescence.

Anti-CD8 staining on paraffin-embedded sections was performed on heart allografts on day 28.

#### T cell transfer experiments.

T cells were isolated using a mouse T cell negative selection kit (STEMCELL Technologies), and purity (>95%) was confirmed by flow cytometry. Adoptive T cell transfer from APOL1 G0 or G1 mice to TCRβ-KO mice was performed followed by full MHC-mismatched skin transplantation (BALB/c to TCR-KO) on the next day. Skin allograft survival, T cell activation, and MLR against allogeneic (BALB/c) and a third party (C3H) were performed.

### Transcriptome analyses

#### Bulk RNA-seq.

Quality control (FastQC, version 0.11.8), adapter sequence trimming, and alignment to the mouse genome was done (mm39). DEGs (limma-voom, version 3.46.0), with a nominal *P* value of less than 0.01, underwent enrichment analysis (enrichR, version 2.1, R package).

#### scRNA-seq analysis.

scRNA-seq was performed by the Center for Cellular Profiling at Brigham and Women’s Hospital (10X Genomics protocol). Samples were identified by the highest expressed hashtag sequence. Unsupervised clustering was conducted with the first 30 principal components (PCs) with a resolution parameter of 0.8. Cell annotation was based on previous publications (Figure 6, D and E). DEG analysis was conducted with Seurat (version 4.1.1) using the Wilcoxon rank-sum test. Sensitivity analysis was conducted by removing the Hashtag5 sample. Downsampling DEG analysis was conducted by randomly choosing the same number of G0 cells from the G1 cell population (equally from each G1 sample) for each cell type. Downsampling was conducted 100 times and DEGs consistently seen (sample direction of fold change) in 50 of the 100 analyses were considered robust DEGs. Enrichment analysis was conducted with “enrichR” (version 2.1) R package.

### Human cohorts

#### Yale CKD cohorts.

We used ongoing studies recruiting patients with CKD: (a) a single-center, prospective observational study of AA individuals from Yale kidney clinics with a clinically confirmed APOL1 genotype, who consented to sample collection; (b) a multicenter, randomized controlled trial involving individuals with FSGS (NCT06090227), in which patients consented to pre-randomization blood collection (17). PBMCs were processed according to the CTOT protocols (30).

#### CHARM cohort.

The details of the longitudinal CHARM study of SARS-CoV-2 infection of US Marine Corps recruits at the Marine Corps Recruit Depot (Parris Island, South Carolina, USA) are reported elsewhere (49). The demographics of the CHARM cohort, Olink data, and peripheral blood RNA-seq data are downloadable from the Gene Expression Omnibus (GEO) database (GEO GSE198449). The APOL1 genotype was evaluated by aligning the RNA-seq reads to the *APOL1* gene.

#### All-of-Us data.

The APOL1 genotypes were extracted from the whole-exome sequencing (WES) results from the All-of-Us biobank (total, *n* = 238,883 [*n* = 94,236 male individuals]; no risk alleles, *n* = 203,43; 1 risk allele, *n* = 28,207; and 2 risk alleles, *n* = 7,241) (50). The disease phenotype was summarized by PheTK based on ICD9 and ICD10 codes (51). Phenotypes with fewer than 50 cases were excluded. Logistic regression on dominant model (1 or 2 risk alleles vs. 0 risk alleles) and additive model (number of risk alleles) was tested by adjusting sex and genetic race (All-of-Us online workbench) (50).

### CTOT-19 study

Details of this trial have been reported elsewhere (Peter S. Heeger, PI; ref. 30).

#### Genotyping of CTOT-19 recipients.

Recipient DNA was genotyped using the Illumina GSA3 chip (*n* = 210). APOL1 risk alleles were directly genotyped. The allele that did not carry G1 or G2 variant was identified as G0 (Supplemental Methods).

#### Flow cytometry CTOT-19.

Surface staining was performed on PBMCs using the previously published CTOT protocols (52). Of the 56 patients with flow data from visit 0, 1 G0/G0 NAR sample, 1 G0/G0 AR sample, 2 APOL1-RV NAR samples, and 1 APOL1-RV AR sample were excluded due to poor staining.

#### Ex vivo human T cell studies.

PBMCs were stimulated for 24 hours with Dynabeads Human T-Activator CD3/CD28 (Gibco, Thermo Fisher Scientific, 11131D) and with Golgi block for intracellular TNF-α and IFN-γ.

### Statistics

An unpaired, 2-tailed *t* test, Mann-Whitney *U* test, or 1-way ANOVA with Tukey’s post hoc test was applied. A *P* value of less than 0.05 was considered statistically significant. For CTOT-19, descriptive statistics were compared using the χ^2^ test and Fisher’s exact test or a 2-tailed *t* test. Kaplan-Meier (K-M) survival curves were calculated with AR as the outcome. Bivariate cox proportional hazard models were developed using APOL1 genotype status and covariates that were significantly different between APOL1-RV and G0/G0 recipients (GraphPad Prism, GraphPad Software). Data are presented as the mean ± SEM.

### Study approval

The studies involving humans were approved by the IRBs of Yale University (New Haven, Connecticut, USA; nos. 2000036180, 2000035723, and 2000030257)) and Mount Sinai Hospital (New York, New York, USA). All participants provided written informed consent. All animal experiments were performed with approval of the IACUCs of Yale University (no. 2023-20495) and Brigham and Women’s Hospital (Boston, Massachusetts, USA) (no. 2019N000153).

### Data availability

Values for all data points are provided in the Supporting Data Values file. The transcriptomics data included in this work are available on the Gene expression Omnibus (GEO) repository (GEO GSE289030 [bulk RNA] and GSE289032 [scRNA]). The programming code is located at https://github.com/ZephyrSun03/APOL1_seq (GitHub commit ID: fcc96c7). Detailed CTOT-19 and CHARM clinical data are given in the respective publications (30, 53). The CTOT-19 recipient genotyping data are available upon request to the corresponding author. MZ-302 availability is restricted and was obtained through a material transfer agreement between Yale University and Maze Therapeutics Inc.

## Author contributions

MCM conceptualized the study. JP, ET, ZS, and MCM were responsible for data curation. JP, ET, ZS, and MCM conducted formal analysis. PSH, JA, SI, and MCM acquired funding. JP, ET, IC, AR, MG, J Choi, SC, HM, RI, BF, IG, JA, NM, and S Smithson performed experiments. SN, XT, S Somlo, SI, and MCM designed the study methodology. Project Administration: JP, ET, HS, and MCM were responsible for project administration. JP, ET, IC, AR, SN, XT, PC, AK, GB, WP, J Craft, JA, NM, SI, PH, and MCM provided resources. IC, MG, BK, WS, JSP, ZZ, J Craft, JA, NM, SI, PH, ASC, and MCM supervised the study. JP, ET, ZS, JA, NM, SI, and MCM. JP, ET, and MCM were responsible for validation of the results. JP, ET, and MCM wrote the original draft of the manuscript. All authors reviewed and edited the manuscript. The order of the co–first authors’ names was determined on the basis of their relative contributions.

## Conflict of interest

The authors have declared that no conflict of interest exists.

## Funding support

This work is the result partly of NIH funding and is subject to the NIH Public Access Policy. Through acceptance of this federal funding, the NIH has been given a right to make the work publicly available in PubMed Central.

- NIH grant R21AI178705 (to MCM and SI)
- Department of Defense (DoD) grant HT94252310441 (to MCM and SI)
- NIH grant dR01DK122164 and R01DK132274 (to MCM)
- DoD grant HT94252310454 (to MCM)
- NIAID, NIH grant T32 AI155387 (to EMT)
- National Cancer Institute (NCI) Cancer Center Support grant no. NIH P30CA016359 (to the Yale Flow Cytometry Core).
- NIH Shared Instrument grant no. S10OD026996 (to the Yale Flow Cytometry Core).

## Supplementary Material

Supplemental data

Unedited blot and gel images

Supplemental tables 2-6

Supporting data values

## Figures and Tables

**Table 3:**
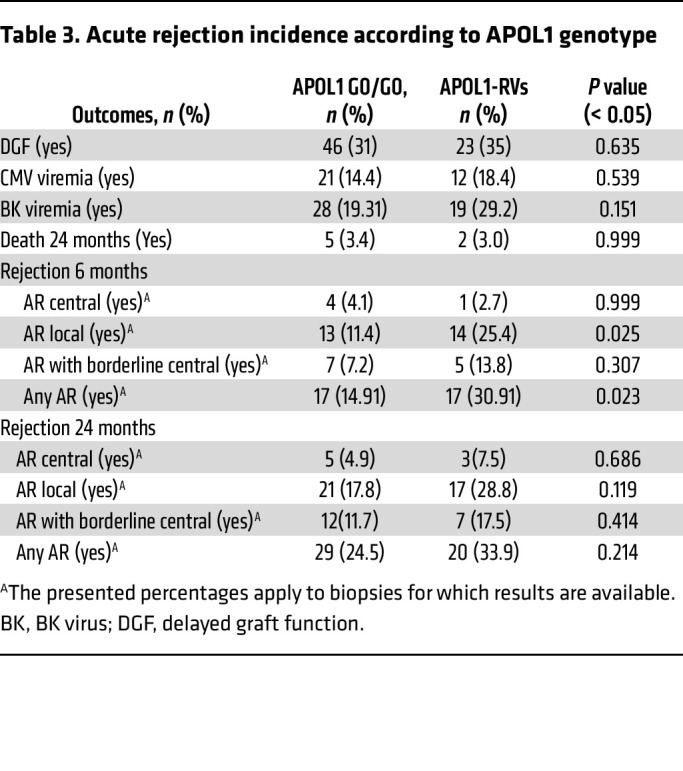
Acute rejection incidence according to APOL1 genotype

**Table 1:**
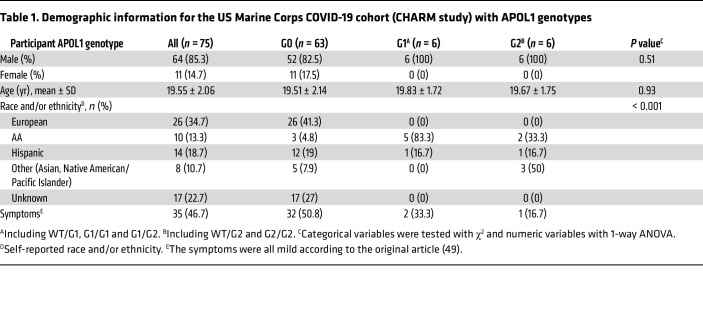
Demographic information for the US Marine Corps COVID-19 cohort (CHARM study) with APOL1 genotypes

**Table 2:**
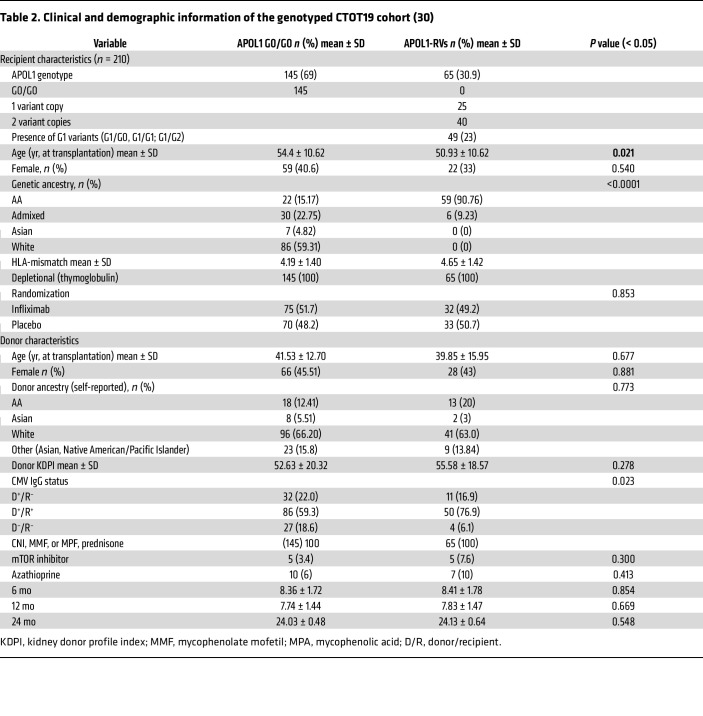
Clinical and demographic information of the genotyped CTOT19 cohort (30)

## Version 1 06/09/2026

In-Press Preview

## Version 2 08/03/2026

Electronic publication

## References

[1] Gbadegesin RA, Ulasi I, Ajayi S, Raji Y, Olanrewaju T, Osafo C (2025). APOL1 Bi- and monoallelic variants and chronic kidney disease in West Africans. N Engl J Med.

[2] Thomson R (2014). Evolution of the primate trypanolytic factor APOL1. Proc Natl Acad Sci U S A.

[3] Genovese G (2010). Association of trypanolytic ApoL1 variants with kidney disease in African Americans. Science.

[4] Beckerman P (2017). Transgenic expression of human APOL1 risk variants in podocytes induces kidney disease in mice. Nat Med.

[5] Gerstner L (2022). Inhibition of endoplasmic reticulum stress signaling rescues cytotoxicity of human apolipoprotein-L1 risk variants in Drosophila. Kidney Int.

[6] Wu J (2021). The key role of NLRP3 and STING in APOL1-associated podocytopathy. J Clin Invest.

[7] McCarthy GM (2021). Recessive, gain-of-function toxicity in an APOL1 BAC transgenic mouse model mirrors human APOL1 kidney disease. Dis Model Mech.

[8] Datta S (2024). APOL1-mediated monovalent cation transport contributes to APOL1-mediated podocytopathy in kidney disease. J Clin Invest.

[9] Vandorpe DH (2023). Apolipoprotein L1 (APOL1) cation current in HEK-293 cells and in human podocytes. Pflugers Arch.

[10] Zimmerman B (2025). Small molecule APOL1 inhibitors as a precision medicine approach for APOL1-mediated kidney disease. Nat Commun.

[11] Freedman BI (2016). APOL1 genotype and kidney transplantation outcomes from deceased African American donors. Transplantation.

[12] Zhang Z (2021). Recipient APOL1 risk alleles associate with death-censored renal allograft survival and rejection episodes. J Clin Invest.

[13] Roy N (2023). Association of recipient APOL1 kidney risk alleles with kidney transplant outcomes. Transplantation.

[14] Zanoni F (2024). Genetic versus self-reported African ancestry of the recipient and neighborhood predictors of kidney transplantation outcomes in 2 multiethnic urban cohorts. Am J Transplant.

[15] Sanghavi K (2017). Genotype-guided tacrolimus dosing in African-American kidney transplant recipients. Pharmacogenomics J.

[16] Song W (2022). Development of Tbet- and CD11c-expressing B cells in a viral infection requires T follicular helper cells outside of germinal centers. Immunity.

[17] Barsotti GC (2024). Rationale and design of a phase 2, double-blind, placebo-controlled, randomized trial evaluating AMP kinase-activation by metformin in focal segmental glomerulosclerosis. Kidney Int Rep.

[18] Zhang Z (2017). APOL1 G2 risk allele-clarifying nomenclature. Kidney Int.

[19] https://www.asn-online.org/education/kidneyweek/2023/program-abstract.aspx?controlId=3944929.

[20] Alexopoulou L (2001). Recognition of double-stranded RNA and activation of NF-kappaB by Toll-like receptor 3. Nature.

[21] Hung AM (2022). APOL1 risk variants, acute kidney injury, and death in participants with African ancestry hospitalized with COVID-19 from the Million Veteran Program. JAMA Intern Med.

[22] Shetty AA (2021). COVID-19-associated glomerular disease. J Am Soc Nephrol.

[23] Zhao J (2021). ACTH treatment promotes murine cardiac allograft acceptance. JCI Insight.

[24] Horwitz JK (2022). Linking erythropoietin to Treg-dependent allograft survival through myeloid cells. JCI Insight.

[25] Stoeckius M (2018). Cell Hashing with barcoded antibodies enables multiplexing and doublet detection for single cell genomics. Genome Biol.

[26] Szabo PA (2019). Single-cell transcriptomics of human T cells reveals tissue and activation signatures in health and disease. Nat Commun.

[27] Inesi G, Sagara Y (1992). Thapsigargin, a high affinity and global inhibitor of intracellular Ca^2+^ transport ATPases. Arch Biochem Biophys.

[28] Ohga K (2008). Characterization of YM-58483/BTP2, a novel store-operated Ca^2+^ entry blocker, on T cell-mediated immune responses in vivo. Int Immunopharmacol.

[29] Collatz MB (1997). Intracellular calcium chelator BAPTA protects cells against toxic calcium overload but also alters physiological calcium responses. Cell Calcium.

[30] Hricik DE (2023). Infliximab induction lacks efficacy and increases BK virus infection in deceased donor kidney transplant recipients: results of the CTOT-19 trial. J Am Soc Nephrol.

[31] Sun Z (2023). Multiscale genetic architecture of donor-recipient differences reveals intronic LIMS1 mismatches associated with kidney transplant survival. J Clin Invest.

[32] Lee BT (2012). The APOL1 genotype of African American kidney transplant recipients does not impact 5-year allograft survival. Am J Transplant.

[33] Zhang Z (2024). Genetic ancestry, proportion of African ancestry, and apolipoprotein L1 risk variants in kidney recipients. Am J Transplant.

[34] Egbuna O (2023). Inaxaplin for proteinuric kidney disease in persons with two *APOL1* variants. N Engl J Med.

[35] Vaeth M (2017). Store-operated Ca^2+^ entry controls clonal expansion of T cells through metabolic reprogramming. Immunity.

[36] Li Y (2005). Role for protein kinase Ctheta (PKCtheta) in TCR/CD28-mediated signaling through the canonical but not the non-canonical pathway for NF-kappaB activation. J Biol Chem.

[37] Vincenti F (2016). Belatacept and long-term outcomes in kidney transplantation. N Engl J Med.

[38] Freedman BI (2020). *APOL1* long-term kidney transplantation outcomes network (APOLLO): design and rationale. Kidney Int Rep.

[39] Lannon H (2019). Apolipoprotein L1 (APOL1) risk variant toxicity depends on the haplotype background. Kidney Int.

[41] Wang S (2009). A new positive/negative selection scheme for precise BAC recombineering. Mol Biotechnol.

[42] Cui C (2021). Neoantigen-driven B cell and CD4^+^ T follicular helper cell collaboration promotes anti-tumor CD8^+^ T cell responses. Cell.

[43] Chernova I (2023). The ion transporter Na^+^-K^+^-ATPase enables pathological B cell survival in the kidney microenvironment of lupus nephritis. Sci Adv.

[44] Demoulins T (2009). Poly (I:C) induced immune response in lymphoid tissues involves three sequential waves of type I IFN- expression. Virology.

[45] Lan X (2015). Protein domains of APOL1 and its risk variants. Exp Mol Pathol.

[46] Menon MC (2015). Intronic locus determines SHROOM3 expression and potentiates renal allograft fibrosis. J Clin Invest.

[47] Borges TJ (2021). Overexpression of PD-1 on T cells promotes tolerance in cardiac transplantation via ICOS-dependent mechanisms. JCI Insight.

[48] Cai S (2020). Donor myeloid derived suppressor cells (MDSCs) prolong allogeneic cardiac graft survival through programming of recipient myeloid cells in vivo. Sci Rep.

[49] Letizia AG (2020). SARS-CoV-2 Transmission among Marine recruits during quarantine. N Engl J Med.

[50] All of Us Research Program Genomics Investigators (2024). Genomic data in the All of Us Research Program. Nature.

[51] Tran TC (2024). PheWAS analysis on large-scale biobank data with PheTK. Bioinformatics.

[52] Fribourg M (2019). T cell exhaustion correlates with improved outcomes in kidney transplant recipients. Kidney Int.

[53] Sedegah M (2022). CHARM: COVID-19 health action response for Marines-association of antigen-specific interferon-gamma and IL2 responses with asymptomatic and symptomatic infections after a positive qPCR SARS-CoV-2 test. PLoS One.

