# Supplementary material for: APOL1 risk alleles modulate T cell receptor signaling to promote allograft rejection
Source: J Clin Invest. 2026 Jun 9;136(15):e193173. doi: 10.1172/JCI193173 (PMC13430025; doi:10.1172/JCI193173)

Whole blots

Main Figure 3

Supplemental Figure S1

Full unedited blots for Figure 3B

APOL1

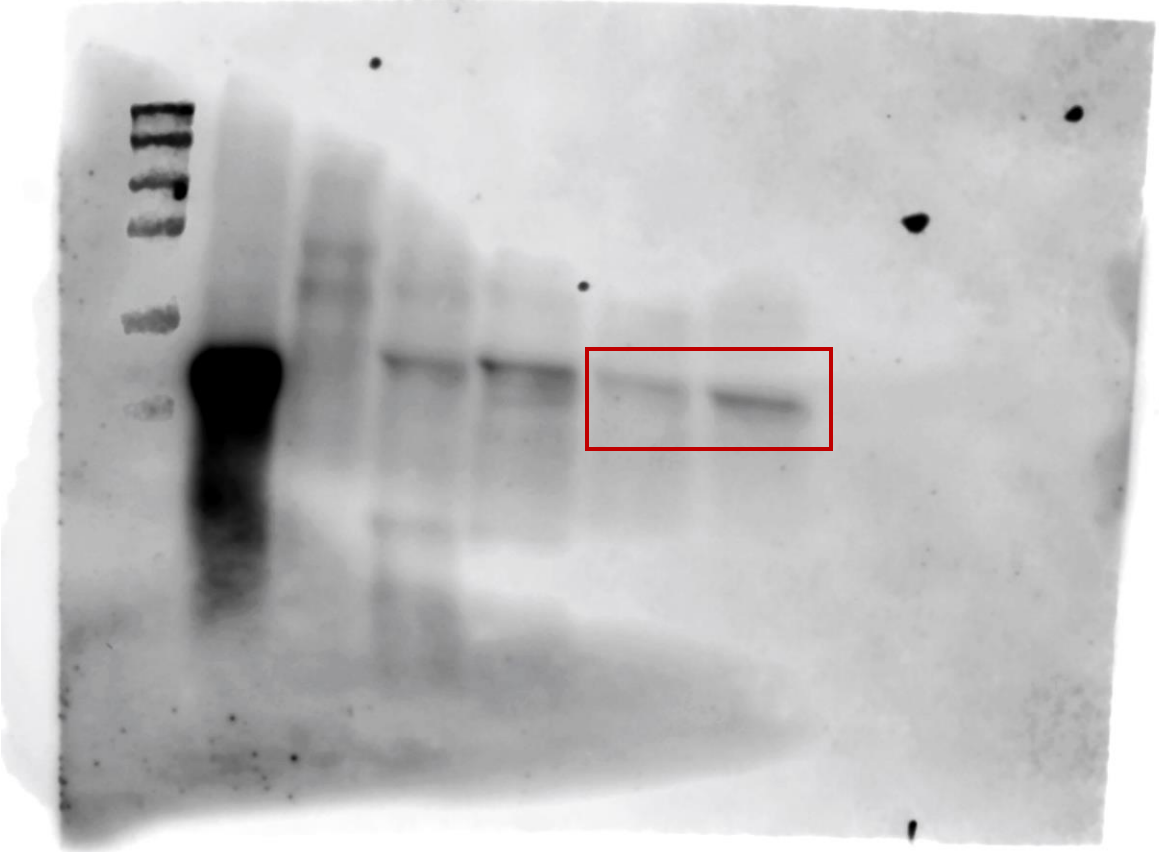

HSP90

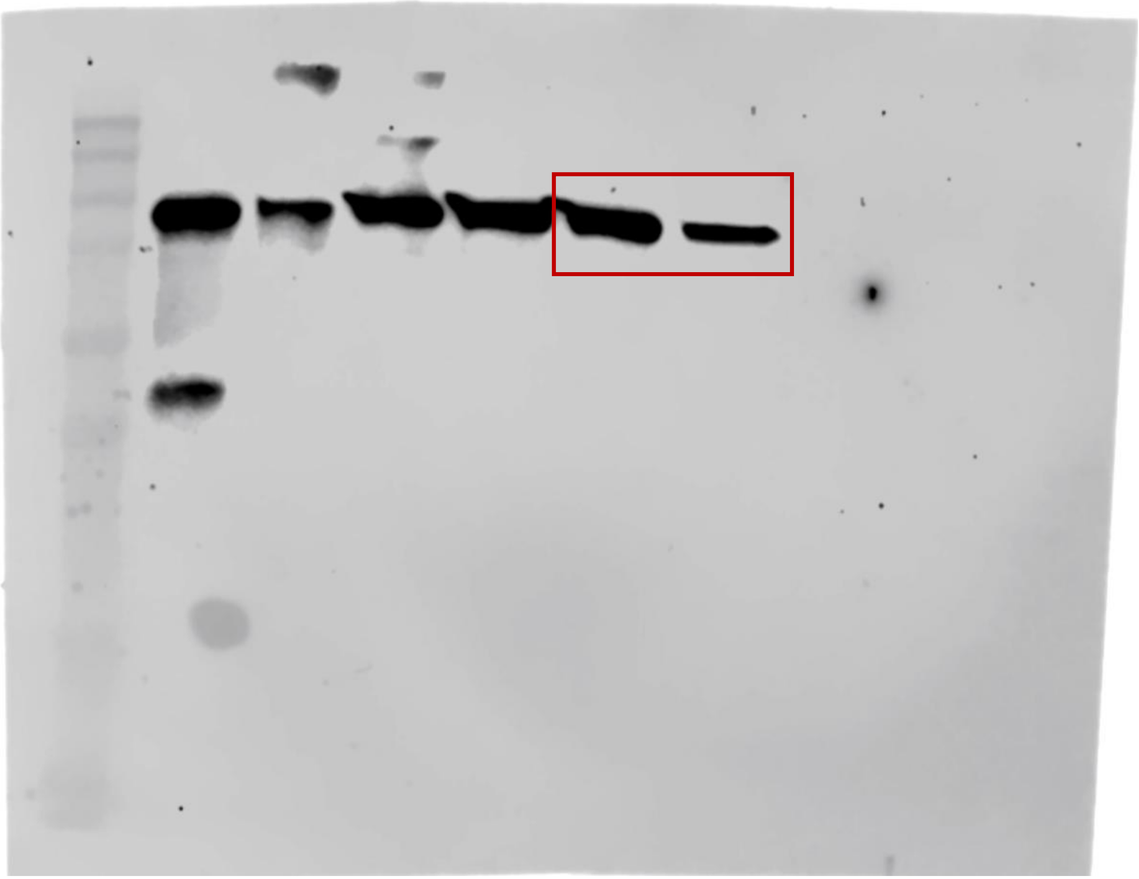

Full unedited blots for Figure 3N

APOL1

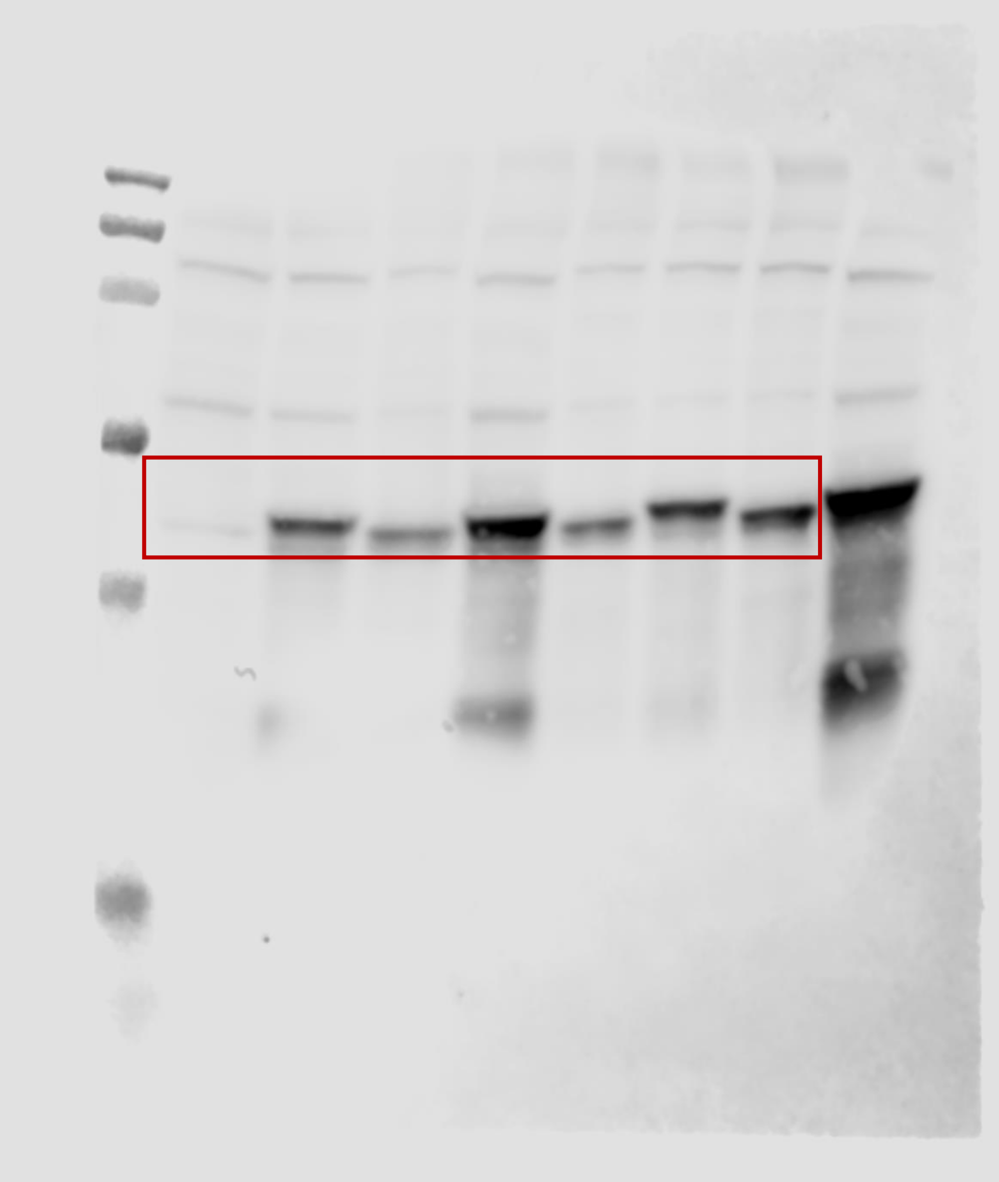

Actin

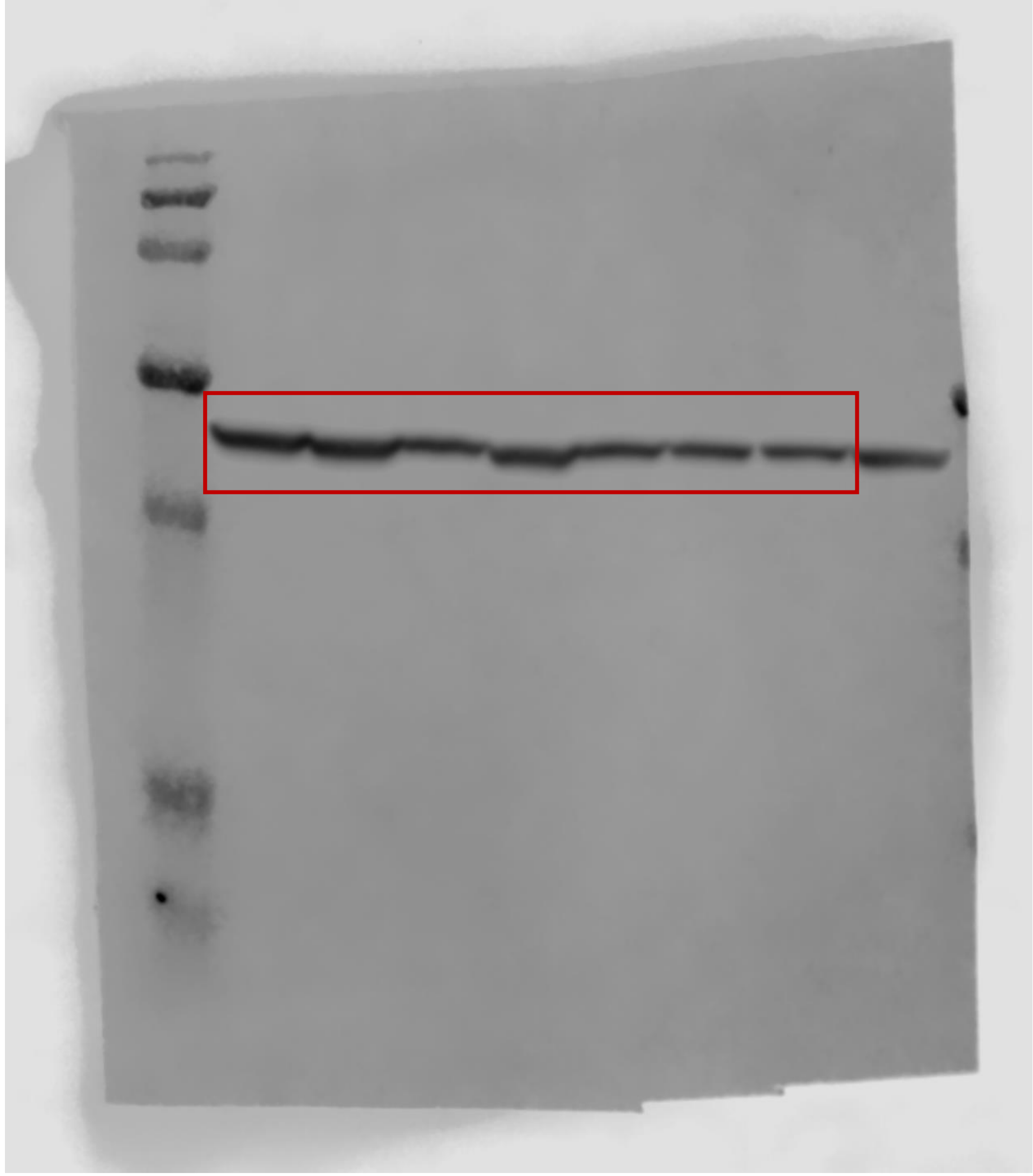

Full unedited blot for Figure 3O

APOL1

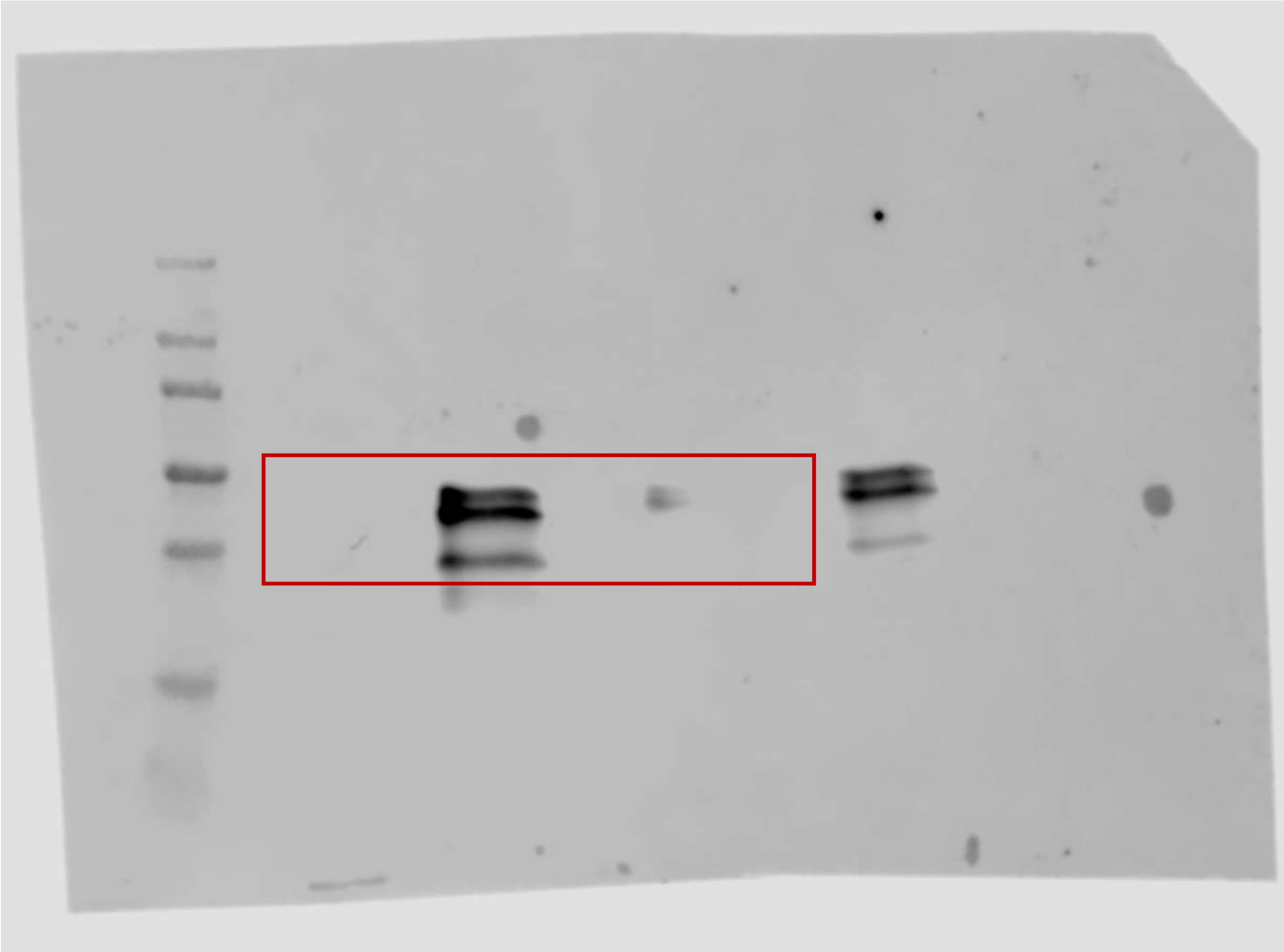

Full unedited blots for Supplemental Figure S1C

APOL1

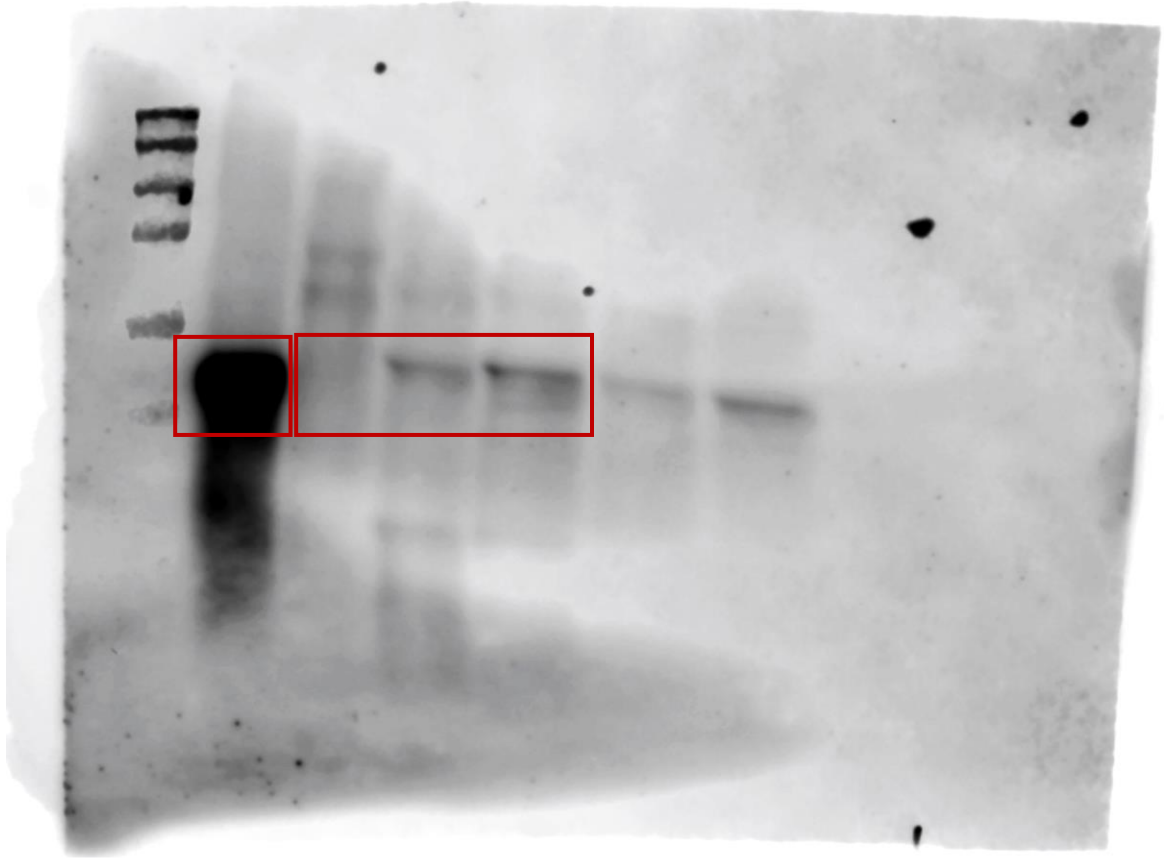

HSP90

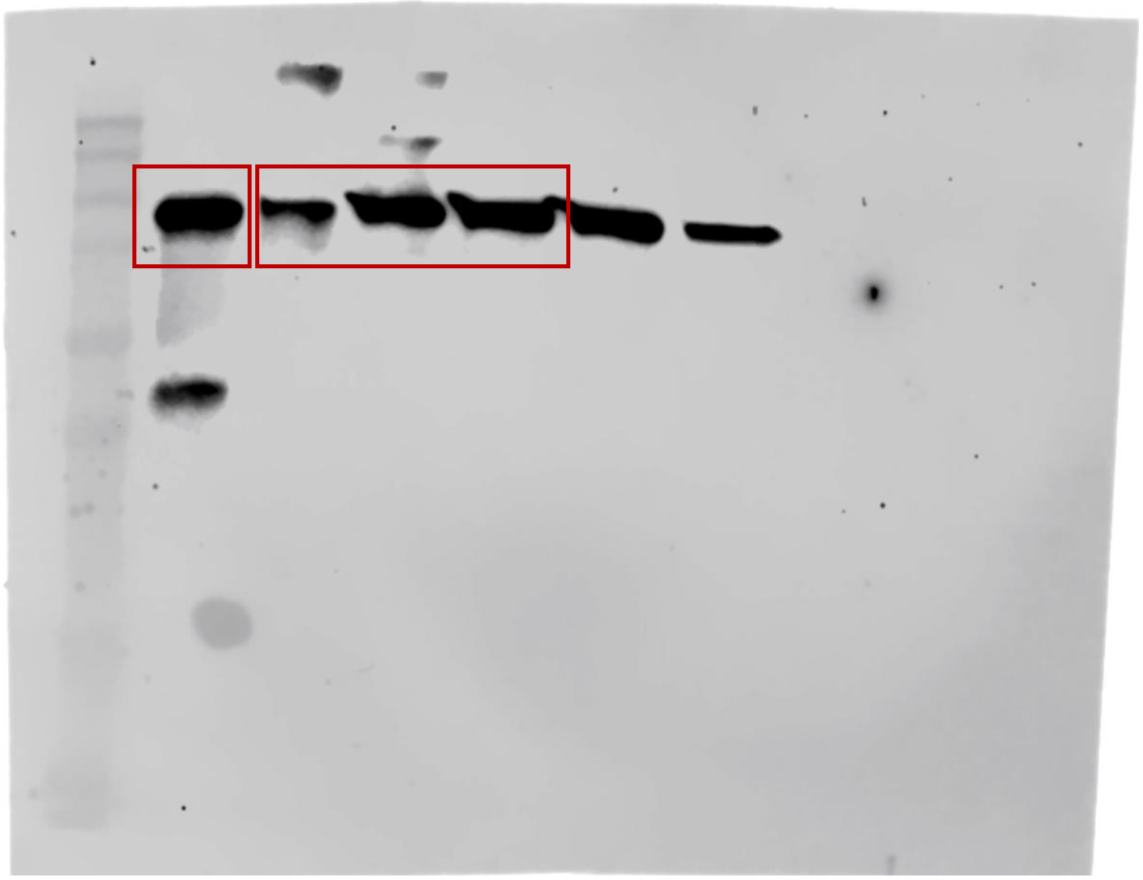

Supplement: Unedited blot and gel images [file jci-136-193173-s119.pdf]
